# Pneumococcal Colonization and Virulence Factors Identified Via Experimental Evolution in Infection Models

**DOI:** 10.1093/molbev/msab018

**Published:** 2021-01-27

**Authors:** Angharad E Green, Deborah Howarth, Chrispin Chaguza, Haley Echlin, R Frèdi Langendonk, Connor Munro, Thomas E Barton, Jay C D Hinton, Stephen D Bentley, Jason W Rosch, Daniel R Neill

**Affiliations:** 1 Department of Clinical Infection, Microbiology and Immunology, Institute of Infection, Veterinary and Ecological Sciences, University of Liverpool, Liverpool, United Kingdom; 2 Parasites and Microbes Programme, Wellcome Sanger Institute, Wellcome Genome Campus, Cambridge, United Kingdom; 3 Department of Infectious Disease, St. Jude Children’s Research Hospital, Memphis, TN, USA

**Keywords:** microbial evolution, pathogens, in-host adaptation, *Streptococcus pneumoniae*, respiratory infection, experimental evolution

## Abstract

*Streptococcus pneumoniae* is a commensal of the human nasopharynx and a major cause of respiratory and invasive disease. We examined adaptation and evolution of pneumococcus, within nasopharynx and lungs, in an experimental system where the selective pressures associated with transmission were removed. This was achieved by serial passage of pneumococci, separately, in mouse models of nasopharyngeal carriage or pneumonia. Passaged pneumococci became more effective colonizers of the respiratory tract and we observed several examples of potential parallel evolution. The cell wall-modifying glycosyltransferase LafA was under strong selection during lung passage, whereas the surface expressed pneumococcal vaccine antigen gene *pvaA* and the glycerol-3-phosphate dehydrogenase gene *gpsA* were frequent targets of mutation in nasopharynx-passaged pneumococci. These mutations were not identified in pneumococci that were separately evolved by serial passage on laboratory agar. We focused on *gpsA*, in which the same single nucleotide polymorphism arose in two independently evolved nasopharynx-passaged lineages. We describe a new role for this gene in nasopharyngeal carriage and show that the identified single nucleotide change confers resistance to oxidative stress and enhanced nasopharyngeal colonization potential. We demonstrate that polymorphisms in *gpsA* arise and are retained during human colonization. These findings highlight how within-host environmental conditions can determine trajectories of bacterial evolution. Relative invasiveness or attack rate of pneumococcal lineages may be defined by genes that make niche-specific contributions to bacterial fitness. Experimental evolution in animal infection models is a powerful tool to investigate the relative roles played by pathogen virulence and colonization factors within different host niches.

## Introduction

Pneumonia and invasive disease caused by *Streptococcus pneumoniae* is a major contributor to global morbidity and mortality, but the primary lifestyle of this opportunistic pathogen is as a commensal of the nasopharynx. Compromised defence mechanisms may render a host permissive to disease, as exemplified by the rising susceptibility to pneumococcal pneumonia in the elderly (Drijkoningen and Rohde 2014), whereas the suite of pneumococcal virulence factors contributes to disease development via evasion of host defences and stimulation of inflammation (Brooks and Mias 2018). Several of these virulence factors are likely retained by the pneumococcus primarily as a means to ensure onwards transmission from nasopharynx, via the stimulation of the inflammation necessary to promote detachment from host surfaces. 

The positive correlation between transmission and virulence is well described for a range of pathogens (Lipsitch and Moxon 1997). Experimental evolution approaches (McDonald 2019) have contributed to this understanding, whereby infectious material is passaged from host to host, in an experimental system that promotes gain of infectivity via selection of individuals displaying high levels of in vivo growth. In studies with viral and parasite species, this approach has demonstrated that increased virulence mirrors the increase in transmissibility (Woolcock and Crighton 1979; Diffley et al. 1987; Dearsly et al. 1990; Scheiblauer et al. 1995).

The functional link between transmission and virulence traits has been demonstrated with pneumococcus in relation to its haemolytic toxin, pneumolysin. Pneumolysin contributes to pathology via direct lysis of host cells and stimulation of inflammation and pneumolysin-deficient mutants of *S. pneumoniae* demonstrate markedly attenuated virulence in animal models (Berry et al. 1989; Hirst et al. 2004; McNeela et al. 2010). Pneumococcal lineages in which pneumolysin lacks pore-forming activity typically cause nonlethal respiratory disease in humans (Jefferies et al. 2007). Zafar and colleagues have demonstrated that the inflammation stimulated by pneumolysin in the upper respiratory tract contributes to shedding of bacteria from the mucosal surfaces and thereby promotes transmission to new hosts (Zafar et al. 2017). In the absence of virus-induced inflammation, which similarly promotes shedding (Zafar et al. 2016), pneumolysin may be the primary means by which pneumococcus ensures onward transmission. However, pneumolysin is detrimental for carriage in the upper airways, as its proinflammatory properties hasten clearance of colonization (Matthias et al. 2008). The trajectory of pneumococcal evolution has thus likely been determined by the need to balance the processes of colonization and transmission.

We sought to determine how pneumococcus might adapt to its natural niche in the nasopharynx when selective pressures associated with transmission were removed. Transmission of *S. pneumoniae* is associated with a single-cell bottleneck (Kono et al. 2016) and so mutations compromising transmission would not be retained in a natural setting, even if they confer advantages in colonization. Therefore, we manually passaged pneumococci between mice in a model of asymptomatic nasopharyngeal carriage. In parallel, we also performed the same process during lung infection, to determine whether environmental differences between the upper and lower airways might shape pneumococcal adaptation and evolution. Our results demonstrate rapid emergence and selection of advantageous polymorphisms in both nasopharynx and lung-passaged pneumococci. Some polymorphisms were common to several independently evolved lineages of pneumococcus, but these examples of potential parallel evolution were found in different genes in nasopharynx versus lung-evolved lineages.

## Results

### In Vivo Experimental Evolution of *S. pneumoniae* in Murine Nasopharynx and Lungs Selects for Increased Niche Colonization Potential

**Fig. 1.**
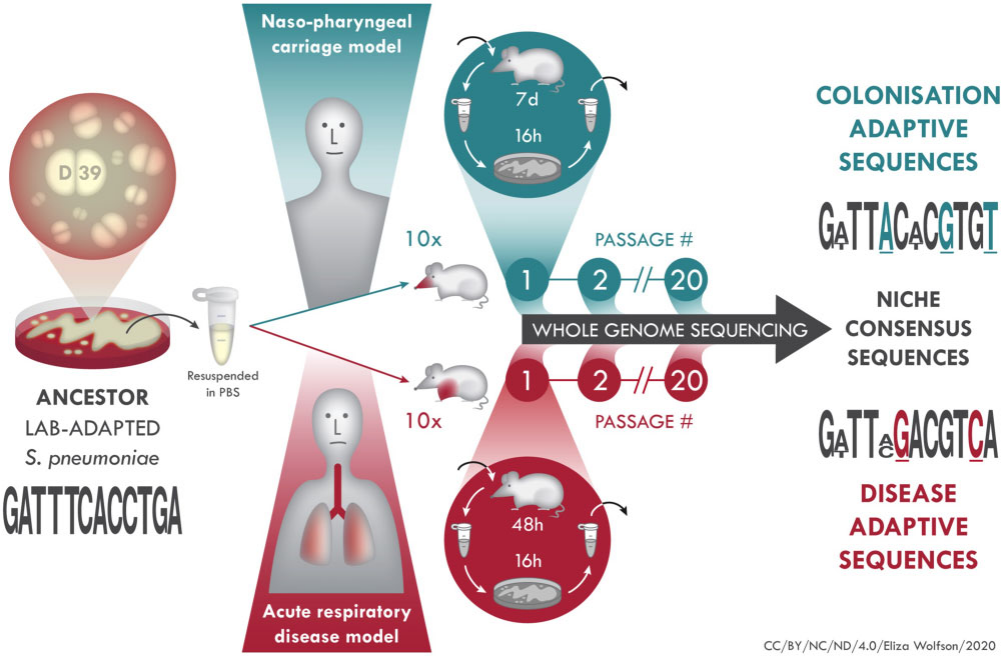
In vivo experimental evolution of *S. pneumoniae*. D39 (ancestor) was grown on agar, colonies resuspended in PBS and mice intranasally infected with 10 µl (carriage model, ten mice) or 50 µl (pneumonia model, ten mice) inoculum. The 20 infected mice were treated as founders of independent pneumococcal lineages. After each passage, which was 7 days for carriage, 48 h for pneumonia, pneumococci were recovered from nasopharynx, in the carriage model, or lungs, in the pneumonia model and, after minimal passage on agar, introduced into a new mouse for the next round of passage. At every passage and at the final (20×) passage, pneumococci were stored for future analysis, including whole genome sequencing for identification of adaptive mutations associated with colonization of nasopharynx or lungs.

To perform experimental evolution of *S. pneumoniae*, we chose the serotype 2 strain D39. Isolated nearly a century ago by Avery, this strain has been widely propagated in a laboratory environment and so may have lost many of its adaptations to the host (Lanie et al. 2007). Using an inoculum prepared from a single colony, we separately infected the nasopharynx or the lung of CD1 mice, infecting 10 mice each per niche. Each of these 20 individuals was considered a founder for an independent experimental evolution lineage of pneumococcus (fig. 1). Thereafter, pneumococci were recovered from either nasopharynx or lungs. After minimal growth on agar, the entire population was used to infect a further mouse for the next round of passage. This process was repeated 20 times per lineage, with 7 days of nasopharyngeal carriage or up to 48 h of lung infection for each round of passage. This yielded 10 nasopharynx-passaged lineages and 10 lung-passaged lineages. Each nasopharynx-passaged lineage had spent 140 days in total in the nasopharynx, whereas the lung-passaged lineages had spent ∼26 days in lungs. To control for bottlenecks or selective pressures induced by growth under laboratory conditions between passages, we also serially passaged pneumococci 20 times on gentamicin agar, transferring a dilution of the entire bacterial population at each passage that was approximately equal to the bacterial numbers recovered from nasopharynx and lungs during passage in the infection models.

**Fig. 2.**
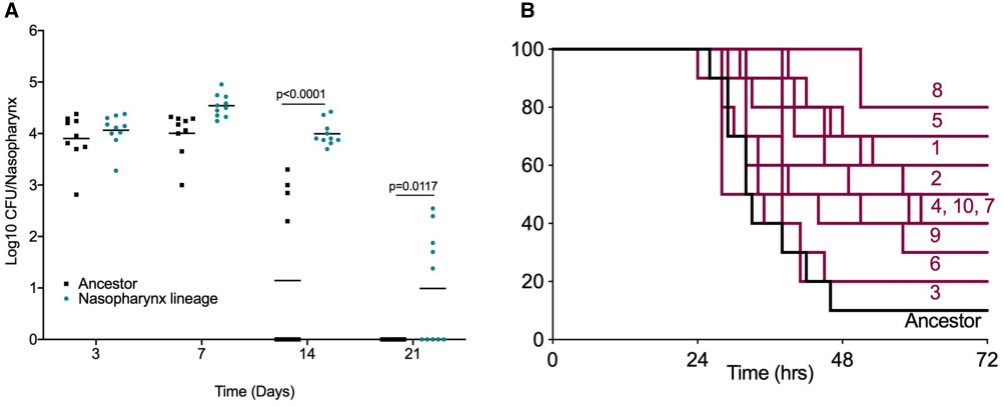
Tissue-passaged pneumococci show evidence of niche adaptation. Mice were infected with the ancestral D39 population or 20 times passaged lineages from (*A*) nasopharyngeal carriage (lineage 1) or (*B*) pneumonia models. (*A*) Mice were infected with 1 × 10^5^ CFU of the ancestor or nasopharynx lineage 1 to induce asymptomatic carriage. Bacterial numbers in nasopharynx were determined by serial dilution of tissue homogenates onto blood agar at days 3, 7, 14, and 21 postinfection. *P*-values are from two-way ANOVA with Sidak’s multiple comparison test. (*B*) Mice were infected with 1 × 10^6^ CFU of the ancestor or one of the ten lung-passaged lineages to induce pneumonia (*n* = 10 per group). Mice were monitored for signs of disease and culled once lethargic. Survival curves were compared by Mantel–Cox log-rank test (table 1). Labels show lineage numbers.

To determine whether in vivo experimental evolution had resulted in altered pneumococcal phenotypes, the 20 times nasopharynx-passaged lineages were assessed for their ability to colonize in a nasopharyngeal carriage model (fig. 2*A* and table 1). Nasopharyngeal colonization density was comparable in the ancestor and a nasopharynx-passaged lineage over the first 7 days of infection, but thereafter clearance of the ancestor proceeded more rapidly (fig. 2*A*). All 10 lineages showed increased colonization prevalence at day 14 postinfection, relative to the ancestor and, in those mice that remained colonized, mean colonization density was higher in all lineages except for lineage 8 (table 1).

**Table 1.** Phenotypes of Nasopharynx-Passaged and Lung-Passaged *Streptococcus pneumoniae* Lineages.

Nasopharynx-Adapted Lineage^a^	Percentage Mice Colonized at 14 Days (*P*-value^b^ vs. Ancestor)	Nasopharynx Colonization Density at 14 Days, Colonized Mice Only (log10 Mean CFU (SD))
Ancestor (D39)	40	2.860 (0.421)
1	*100* (*0.004)	3.995 (0.234)
2	*90* (*0.022)	3.549 (0.651)
3	80 (0.075)	3.484 (0.890)
4	*90* (*0.022)	3.086 (0.804)
5	80 (0.075)	2.928 (0.632)
6	*90* (*0.022)	3.418 (0.485)
7	80 (0.075)	4.138 (0.882)
8	50 (0.661)	2.818 (0.596)
9	*100* (*0.004)	3.134 (0.653)
10	70 (0.189)	3.360 (0.880)

Lung-Adapted Lineage^c^	Percentage Mice Surviving to 72 h (*P*-value^d^ vs. ancestor)	Percentage Surviving Mice Still Colonized at 3 Days (log10 mean CFU)

Ancestor (D39)	10	0 (0)
1	*60* (**0.0033)	100 (5.124)
2	*50* (**0.0037)	100 (3.622)
3	10 (0.8706)	100 (5.115)
4	*40* (*0.0185)	25 (0.781)
5	*70* (**0.0027)	57 (1.830)
6	20 (0.5989)	100 (3.975)
7	40 (0.0669)	75 (3.325)
8	*80* (****0.0001)	75 (2.275)
9	30 (0.1213)	33 (0.722)
10	40 (0.0966)	50 (2.144)

a Nasopharynx-passaged lineages were compared in a carriage infection model. *N* = 10 per group.

b *N* − 1 Chi-squared test (carriage proportions) versus the ancestor.

c Lung-passaged lineages were compared in a pneumonia infection model. *N* = 10 per group.

d Mantel–Cox log-rank test versus the ancestor.

Italics denote significant differences as determined by N-1 Chi-squared test (colonization data) or Mantel-Cox log-rank test (survival data).

Next, the 20 times passaged lung lineages were assessed for their ability to colonize murine lungs in a pneumonia model (table 1 and fig. 2*B*). Disease progression was slower in mice infected with lung-passaged lineages in comparison to those infected with the ancestor strain. Survival proportions were increased in mice infected with any of the ten lineages, relative to the ancestor, with significantly different survival curves for lineages 1, 2, 4, 5, and 8 (table 1 and fig. 2*B*). A proportion of surviving mice from each of the infections performed with lung-passaged lineages retained viable pneumococci in lungs, whereas none was recovered from surviving mice infected with the ancestor (table 1).

We determined growth in liquid culture, adhesion to airway epithelial cells *in vitro* and production of pneumolysin in all lineages (supplementary figs. 1–3, Supplementary Material online). Growth dynamics were comparable to that of the ancestor for all lineages, with the exception of lung-passaged lineages 7 and 8, and nasopharynx-passaged lineages 2 and 7, which displayed modestly reduced growth rates in nutrient broth (supplementary figs. 1*A* and *B*, 2, and 3, Supplementary Material online). The levels of adhesion to A549 airway epithelial cells were highly variable in lung-passaged lineages (supplementary fig. S1*C*, Supplementary Material online), but three of the nasopharynx-passaged lineages showed evidence of increased adhesion potential (supplementary fig. S1*D*, Supplementary Material online). Levels of pneumolysin, in lysates prepared from mid-log phase pneumococci, were significantly reduced in five of ten lung-passaged lineages and nine of ten nasopharynx-passaged lineages, relative to the ancestor strain (supplementary fig. 1*E* and *F*, Supplementary Material online). The level of epithelial cell adhesion was negatively correlated with the amount of pneumolysin produced in lung passaged lineages (*R*^2^ = 0.70) (supplementary fig. 1*G*, Supplementary Material online).

### Genomic Characterization of Niche-Passaged Pneumococci

To uncover the genetic basis of the observed adaptations, we genome sequenced 100 pneumococcal populations, which included all 20 times passaged lineages and passage numbers 1, 5, 10, and 15 of each lineage. By comparison with the genome sequence of the ancestor strain, we identified variants that had arisen during experimental evolution. We first performed *de novo* genome assembly for our ancestor D39, using a combination of long- and short-read sequencing. This yielded a single contig genome of 2,046,551 base pairs, a size similar to that determined in a recently published assembly for this strain (Slager et al. 2018). Genome annotation with Prokka (Seemann 2014) identified 1,998 protein-coding sequences, with 664 of these being hypothetical proteins. Deep sequencing of the ancestor population (300× coverage) was performed to enable us to detect low-frequency variants in the inoculum used to establish the 20 experimental evolution lineages. Using Breseq (Deatherage and Barrick 2014), we mapped the Illumina reads from the ancestor inoculum against the consensus genome sequence. After filtering out artefactual or unreliable mutations and those at <2% frequency (the suggested frequency-cut off limit for polymorphism mode in Breseq (Deatherage and Barrick 2014)), we observed 43 unique variants, at frequencies of between 2.2 and 20.7% in the total ancestor population (supplementary data set 1, Supplementary Material online). Most of these were subsequently lost during in vivo experimental evolution. Those that were retained (in genes *dnaK* and *D39N_01974*) are highlighted below.

Variant calling of Illumina reads from in vivo passaged pneumococci, with an average of 147× coverage (range 105×–190×), detected a total of 735 variants across all the nasopharynx lineages (passages 1, 5, 10, 15, and 20), with 206 variants identified in the final populations that had been passaged 20 times through nasopharynx (supplementary data set 1, Supplementary Material online). Within lung-passaged lineages, we observed 509 variants, with 128 found across the 10 populations at passage 20 (supplementary data set 1, Supplementary Material online). The majority of variants detected were single nucleotide polymorphisms (SNPs), although deletions of between 1 and 53 base pairs were identified, as well as several single nucleotide insertions. Indels were predominantly located in intergenic regions. We also identified variants in the control passage lineages that had been generated by serial passage on gentamicin agar. These largely differed from those identified in the nasopharynx and lung lineages (supplementary data set 1, Supplementary Material online).

Cluster of orthologous gene (COG) analysis (Tatusov et al. 2000) of variant data demonstrated that genes with roles in amino acid and nucleotide metabolism accumulated mutations in lineages from both nasopharynx and lungs. In lung-passaged lineages, 16% of variants were found in genes associated with carbohydrate transport and metabolism, whereas 6% of variants in nasopharynx-passaged lineages were in genes associated with energy production and conversion (supplementary fig. 4, Supplementary Material online).

**Fig. 3.**
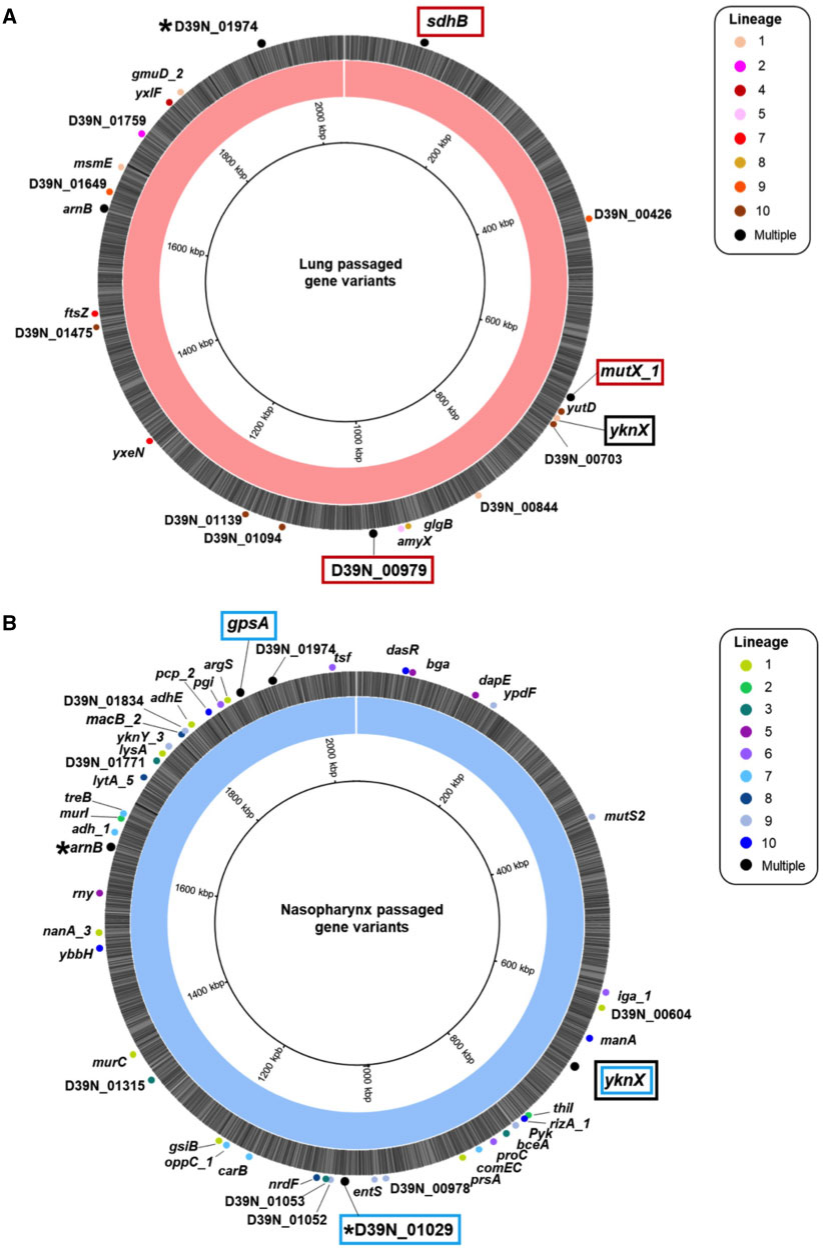
Fixed nonsynonymous mutations identified in 20 times passaged *S. pneumoniae* lineages evolved in mouse lungs or nasopharynx. Genome maps of the D39 ancestor assembly, highlighting the nonsynonymous single nucleotide polymorphisms identified in 20 times passaged pneumococci from the lung (*A*) or nasopharynx (*B*). Labels show the name and position of the gene in which the SNP occurs, with the circles colored according to the lineage where the variant was identified. Black indicates SNPs identified in multiple lineages. Potential examples of parallel evolution are highlighted in red boxes for the lung and blue boxes for the nasopharynx evolved lineages. The same SNP in the *yknX* gene was identified in two nasopharynx lineages, with a separate SNP identified in one lung lineage. This gene is highlighted with a black box. The mutations for all genes are defined in supplementary data set 1, Supplementary Material online, and *’s indicate those genes where different mutations were observed in separate lineages. Variants in *arnB* and *D39N_ 01974* were also identified in the ancestor and control passage.

To identify polymorphisms most likely to drive adaptive phenotypic change, we focused on substitutions that became fixed in the population during experimental evolution. Considering only the final populations in each lineage (that is, those had been passaged 20 times through a niche), we identified 47 different nonsynonymous SNPs within nasopharynx-passaged pneumococci (fig. 3*A*), and 31 in lung-passaged pneumococci (fig. 3*B*), that became fixed (reached 100% frequency) in at least one lineage. In total, 11 nonsynonymous SNPs became fixed in control passage lineages (supplementary fig. 5 and data set 1, Supplementary Material online). The SNPs identified in lung- and nasopharynx-passaged lineages included several examples of potential parallel or convergent evolution, whereby the same polymorphism, or closely located substitutions, arose and reached fixation in two or more independently evolved lineages (table 2). Two of these were observed only in nasopharynx-passaged lineages, three were observed only in lung-passaged lineages and three were found in lineages from both nasopharynx and lungs. Despite the notable changes in pneumolysin production observed, none of the lung or carriage lineages had any variants in the *ply* gene or flanking regulatory regions. Of the SNPs in table 2, the *D39N_01974* polymorphism was identified in both the ancestor population and 6/8 control passage lineages, and one of the four identified *arnB* substitutions (A96V) was found in 7/8 control passage lineages. All SNPs in table 2 that were present in lung or nasopharynx lineages but were not found in the ancestor or control passage were subsequently confirmed by Sanger sequencing.

**Table 2.** Parallel or Convergent Evolution in Niche-Passaged Pneumococcal Lineages.

Gene (Amino Acid Change Caused by SNP)^a^	D39V Annotation^b^	In Passage 20 Nasopharynx Lineages? (*n*/10)	In Passage 20 Lung Lineages? (*n*/10)	In Control Passage Lineages or Ancestor?	COG Category
*mutX1* (P29S)	*SPV_0644* MutT/Nudix Family Protein		2	N	Nucleotide Transport and Metabolism
*D39N_00979* (Y331C)	*SPV_0961 lafA*		3	N	Cell wall/membrane biogenesis
*sdhB* (G14R)	*SPV_0103 sdhB*		2	N	Amino acid transport and metabolism
*arnB* (S192F, A197G, A96V, P11S)	*SPV_1619 aatB*	3	2	Y (A96V)	Amino acid transport and metabolism
*D39N_01974* (A608S, L170M)	*SPV_1969* Glycosyl hydrolase-related protein	3	3	Y	Carbohydrate transport and metabolism
*yknX* (S331N) (L333V)	*SPV_0686* Periplasmic component of efflux system	2	1	N	Cell wall/membrane biogenesis
*D39N_01029* (T62I, Y19H)	*SPV_0912 pvaA*	2		N	Cell wall/membrane biogenesis
*gpsA* (G208R)	*SPV_1918 gpsA*	2		N	Lipid transport and metabolism

a All genes in which nonsynonymous single nucleotide polymorphisms were identified in more than one independently evolved lineage are shown.

b The corresponding gene in the D39 genome was recently published by Slager et al. (2018).

### Evidence of Parallel Evolution in Niche-Passaged Pneumococci

**Fig. 4.**
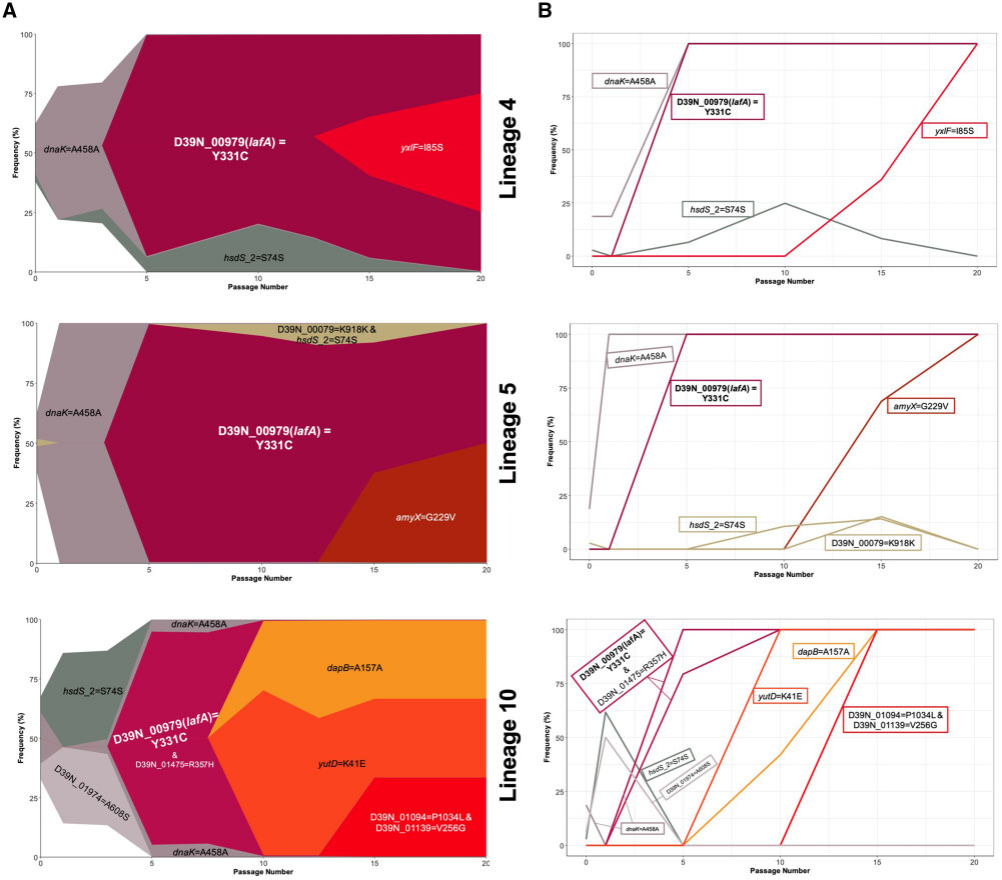
Success of *lafA* polymorphisms in lung-passaged pneumococcal lineages. (*A*) Muller plots showing genotype ancestry and frequency for lineages 4, 5, and 10 of lung-passaged pneumococci. The genotype associated with the *D39N_00979* (*lafA*) SNP is shown in maroon. For lineages 4 and 5, this genotype is uniquely associated with this SNP, whereas in lineage 10 the genotype is also defined by a nonsynonymous mutation in *D39N_01475*. Additional colors are arbitrary but other successful genotypes containing nonsynonymous SNPs are labelled. The ancestral genotype is in white. (*B*) Genotype frequency plots. Where multiple variants define a genotype, order of acquisition is uncertain.

Three lineages of lung-passaged pneumococci acquired the same SNP in *D39N_00979*, encoding the glycosyltransferase LafA, involved in synthesis of lipid anchors for cell wall teichoic acid (Engholm et al. 2017). This SNP arose early in lung lineages 4, 5, and 10 and quickly reached fixation within the population, as can be seen in Muller plots, depicting genotype ancestries, and in genotype frequency plots (fig. 4*A* and *B*). This SNP was not detected in the ancestor or any of the nasopharynx or control passage lineages, suggesting it could confer an advantage within the lung.

**Fig. 5.**
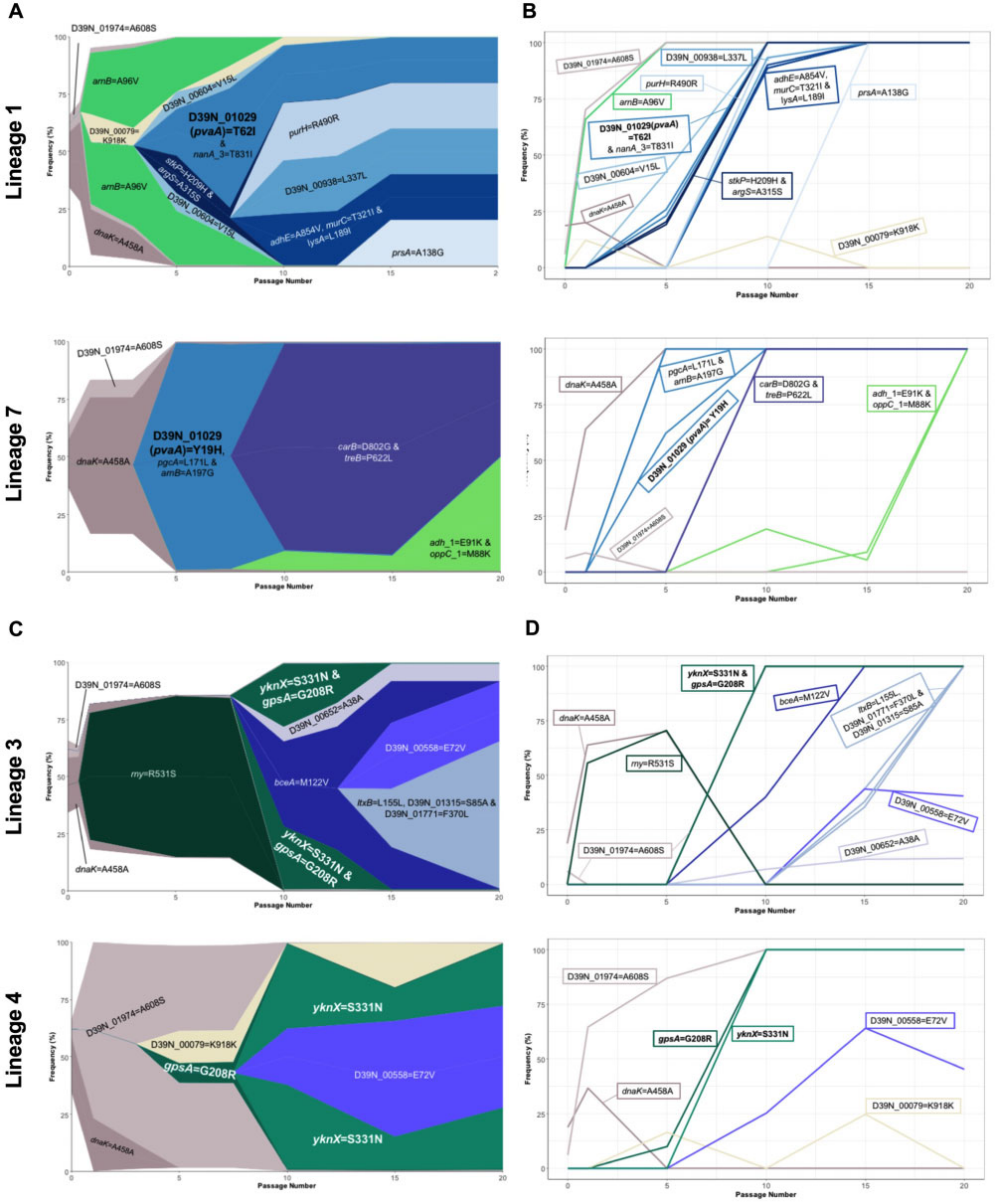
Parallel evolution in nasopharynx-passaged pneumococci. (*A*) Muller plots showing the emergence and success of *D39N_01029* (*pvaA*) variants in nasopharynx-passaged lineages 1 and 7. The *pvaA-*associated genotype is shown in teal. Additional colors are arbitrary but other successful genotypes or those originating from the ancestor population are labelled. (*B*) Genotype frequencies for lineages 1 and 7. (*C*) Muller plots showing the emergence and success of *gpsA* and *yknX* variants in nasopharynx-passaged lineages 3 and 4. The *gpsA* and *yknX* genotypes are shown in shades of green. The two variants are associated with a single genotype in lineage 3. A shared nonsynonymous variant in *D39N_00558* is shown in purple. Additional colors are arbitrary. (*D*) Genotype frequencies for lineages 3 and 4.

For nasopharynx-passaged lineages, two examples of potential parallel evolution were found in the substitutions in *D39N_01029* found in lineages 1 and 7, and the co-occurring *gpsA* and *yknX* SNPs in lineages 3 and 4. Lineages 1 and 7 each acquired a different polymorphism in *D39N_01029* but both resulted in an amino acid change, at positions 62 and 19, respectively (fig. 5*A* and *B*). This gene encodes the surface-expressed pneumococcal vaccine antigen A (*pvaA*), identified in an immunizing screen of pneumococcal proteins as affording protection against disseminated infection (Wizemann et al. 2001). Hava and Camilli (2002) highlighted this gene as being essential for lung infection. Carriage lineages 3 and 4 both acquired the same nonsynonymous SNPs in *gpsA*, encoding a glycerol-3-phosphate dehydrogenase, and *yknX*, encoding a component of an uncharacterized efflux pump. These SNPs became fixed in the populations (fig. 5*C*).

Two lung-passage lineages acquired the same nonsynonymous mutation in the DNA repair gene *mutX1*, conferring a P29S substitution. Both lineages demonstrated an increased frequency of spontaneous rifampicin resistance mutations when grown on antibiotic agar, relative to both the ancestor and to nasopharynx and lung lineages lacking DNA repair gene mutations (supplementary fig. 6 and table 1, Supplementary Material online). Similarly, nasopharynx passaged lineage 9, with a nonsynonymous mutation in *mutS2*, had an increased frequency of spontaneous mutation, consistent with a hypermutator phenotype resulting from defects in DNA repair. Muller plots, genotype ancestries, and genotype frequency plots for all lung and nasopharynx-passaged lineages can be found in supplementary figs. 7–16, Supplementary Material online.

To determine whether observed parallel changes were driven by selective pressures imposed by antibiotic within agar during experimental passage, we measured the minimum inhibitory concentration (MIC) of gentamicin required to inhibit 50% of growth of the ancestor, the lung and nasopharynx passaged lineages and the control passage lineages (supplementary table S2, Supplementary Material online). The MIC50 for the ancestor was 8 µg/ml, and there was evidence of modestly increased resistance to gentamicin amongst the control lineages that had been passaged exclusively on agar, with an MIC50 of 16 µg/ml determined for five lineages. However, all lung- or nasopharynx-passaged lineages had MIC50s of between 4 and 8 µg/ml, suggesting any selection for resistance during growth on agar was offset by subsequent in vivo passage.

**Fig. 6.**
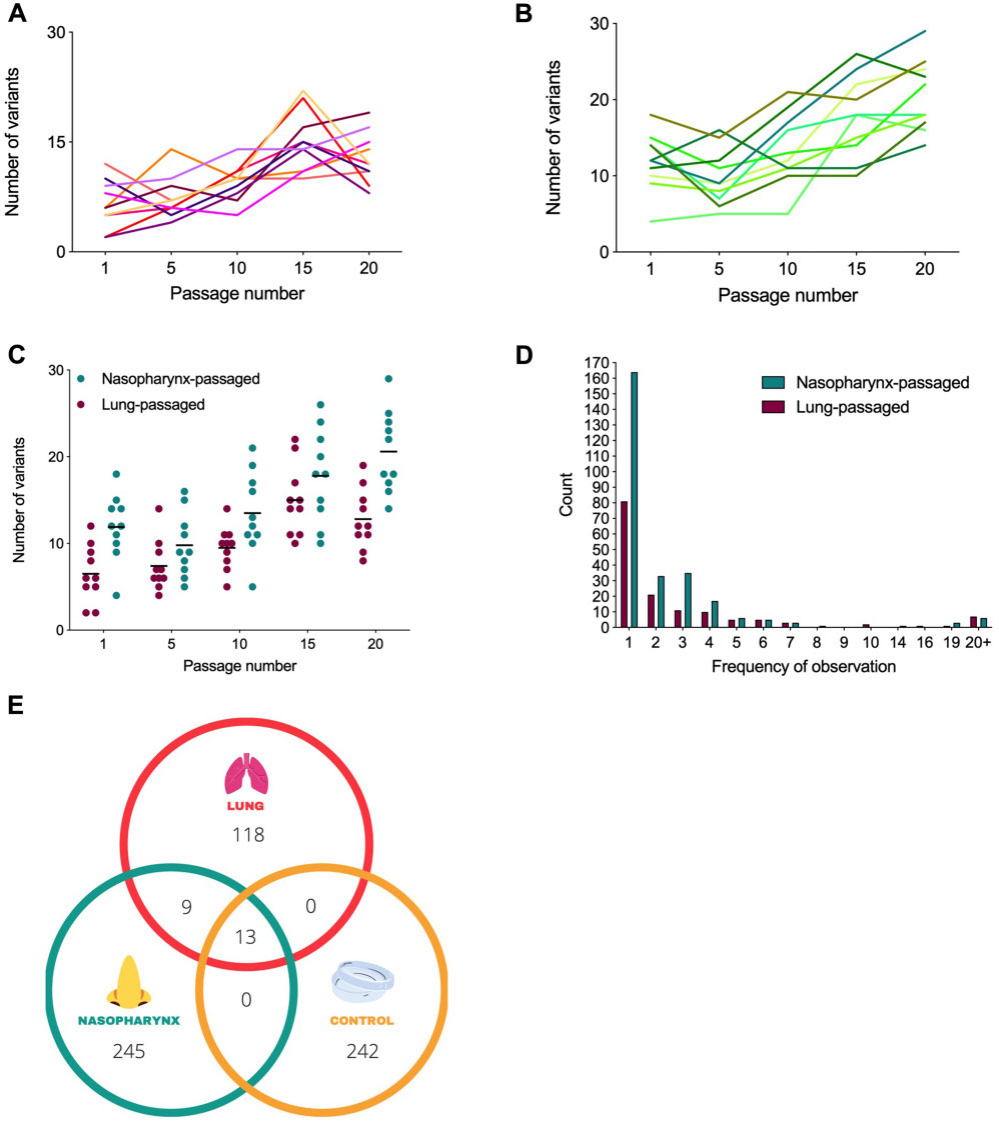
Variant dynamics in niche-passaged pneumococci. (*A*) Number of variants identified per passage in ten *S. pneumoniae* lineages during passage through murine lungs in a pneumonia model. Each line represents an independently evolving lineage (*n* = 1 per lineage, per timepoint). (*B*) Variants per passage for ten independently evolving nasopharynx-passaged lineages in a murine carriage model (*n* = 1 per lineage, per timepoint). (*C*) Comparison of variant numbers per passage in nasopharynx and lung passaged pneumococci. Each dot represents data from a single lineage. Data are a composite of those in (*A*) and (*B*) and are included for clarity. (*D*) Number of times each unique variant was detected. Data are from a composite of all passages of all lineages. For example, if the same variant is detected at passages 1 and 5 in lineage 2 and at passage 20 in lineage 3, but is not found in other lineages, it would be recorded as a count of 3. (*E*) Venn diagram showing unique variants in nasopharynx-, lung-, or lab (control) passaged lineages. Here, each variant is counted only once, regardless of the number of times it was detected.

Several common trends were observed amongst lineages. Between 2 and 22 distinct variants were detected per lineage, per passage, in pneumococci from lungs (fig. 6*A*). Between 4 and 29 distinct variants were detected per lineage, per passage in nasopharynx-passaged pneumococci (fig. 6*B*). Both lung and nasopharynx-passaged lineages had increasing variant numbers in later passages (*P < *0.0001 at passage 15 vs. passage 1, *P = *0.0057 at passage 20 vs. passage 1 for lung lineages, *P =* 0.0113 at passage 15 vs. passage 1, *P < *0.0001 at passage 20 vs. passage 1 for nasopharynx lineages) (fig. 6*C*). The mean number of variants detected was lower at each passage in lung lineages versus nasopharynx, reflecting their reduced time spent in vivo and the effects of population bottlenecks associated with immune clearance mechanisms. Most variants identified were detected only once, that is, in a single lineage at a single passage. However, there was evidence of selection for some SNPs, with 10 appearing ≥10 times in nasopharynx lineages and eleven appearing ≥10 times in lung lineages (fig. 6*D*). Of the total unique variants identified, discounting those in the ancestor population, 245 were found only in nasopharynx lineages and 118 were found only in lung lineages (fig. 6*E*). Within the 22 variants shared between nasopharynx and lung populations, 13 were also in control passage lineages.

### Comparison of Nasopharyngeal Adaptation in Pneumococci from Mouse and Human

A recent longitudinal study assessed microevolution of pneumococcus over 12 months in colonized infants (Chaguza et al. 2020). We mapped sequence reads of pneumococci from this infant study against our ancestor D39 genome. In total, 20 (43%) of the genes in which we observed fixed nonsynonymous SNPs in nasopharynx-passaged lineages were also found to acquire polymorphisms during prolonged colonization in infants (table 3). These included *gpsA* and *yknX*, in which we observed examples of potential parallel evolution (fig. 5*C* and *D*). Considering all genes in which we identified variants (synonymous or nonsynonymous SNPs or indels) in nasopharynx-passaged lineages, 39% (68/174) (supplementary data set 2, Supplementary Material online) were identified in the data set of Chaguza and colleagues. Of note, was *gpsA*, in which SNPs were observed at a frequency of 35.36 per kilobase pair in human colonization, placing it 23rd out of 592 genes in which SNPs were detected, in terms of mutation frequency, and 7th out of 15 genes in which nonsynonymous mutations were identified (Chaguza et al. 2020). These nonsynonymous SNPs mapped to amino acid positions 160, 169, 315, and 322 within GpsA (supplementary fig. 17, Supplementary Material online). BLAST alignment of bacterial peptide sequences of the glycerol-3-phosphate dehydrogenase (G3PDH) product of *gpsA* suggests that amino acid variation is tolerated at most of these positions (supplementary fig. 18, Supplementary Material online). Chaguza et al. identified a substitution of negatively charged aspartic acid for polar asparagine at position 160. The aspartic acid at this position, immediately adjacent to the start of a predicted alpha helix, is largely conserved amongst streptococci, but polar amino acids are present in *Enterococcus faecalis* and *Bacillus subtilis*. The identified substitution at position 169 in a serotype 6B pneumococcus, occurring within a predicted alpha helix, changes a lysine to a glutamic acid. Both amino acids have previously been reported at this position in pneumococci. A histidine to glutamine substitution was reported at amino acid 315, and glutamine has been identified at this position in *Streptococcus sanguinis*, *S. gordonii*, *and S. suis*. The substitution of alanine for aspartic acid at position 322 affects a more highly conserved residue. Hydrophobic alanine or polar serine predominate at this position among pneumococci and related species. The occurrence and apparent selection of *gpsA* variants during natural colonization, together with the observation of a guanine to adenine substitution in *gpsA*, resulting in a glycine to arginine transition at amino acid 208, in nasopharynx lineages 3 and 4 from this study, led us to further investigate the function of this gene during colonization.

**Table 3.** Comparison of SNPs Found in Mouse Nasopharynx-Passaged Lineages and Human Carriage in Infants.

Gene^a^	Present inControl Passage?	Number of SNPs/Episode/Million BasePairs in Human Carriage^b^	Gene Product
*gpsA*	−	216.9537286	Glycerol-3-phosphate dehydrogenase [NAD(P)+]
*D39N_01974*	+	123.8729194	Hypothetical protein
*D39N_01052*	−	63.58701412	Hypothetical protein
*comEC*	−	51.99126547	ComE operon protein 3
*thiI*	−	50.45205037	Putative tRNA sulfur transferase
*D39N_00558*	−	48.30684508	Hypothetical protein
*rny*	−	41.78616878	Ribonuclease Y
*nanA_3*	−	20.02274584	Sialidase A
*iga_1*	−	18.51819576	Immunoglobulin A1 protease
*bceA*	−	16.14465612	Bacitracin export ATP-binding protein BceA
*adh_1*	−	11.8093731	Alcohol dehydrogenase
*yknX*	−	10.21643518	Putative efflux system component YknX
*arnB*	+	9.991806718	UDP-4-amino-4-deoxy-l-arabinose—oxoglutarate aminotransferase
*pacS*	−	8.198177272	putative copper-transporting ATPase PacS
*pflA*	−	7.707247896	Pyruvate formate-lyase-activating enzyme
*manA*	−	6.48516842	Mannose-6-phosphate isomerase ManA
*tsf*	−	5.887686492	Elongation factor Ts
*dapE*	−	4.461795873	Succinyl-diaminopimelate desuccinylase
*pyk*	−	4.070981636	Pyruvate kinase
*carB*	−	1.930449756	Carbamoyl-phosphate synthase large chain

a Genes shown are those with fixed SNPs, identified in 20 times nasopharynx passaged populations from mice and which were also found to be targets for mutation in human carriage.

b From re-analysis of the data set of (2020). Frequencies of mutation per gene per episode of colonization, normalised for gene length (per million base pairs). Episodes of colonization lasted ∼4.44 weeks (mean 7.30, range 1–48).

### Function of *gpsA*, a Gene Associated with Adaptation to the Nasopharynx

**Fig. 7.**
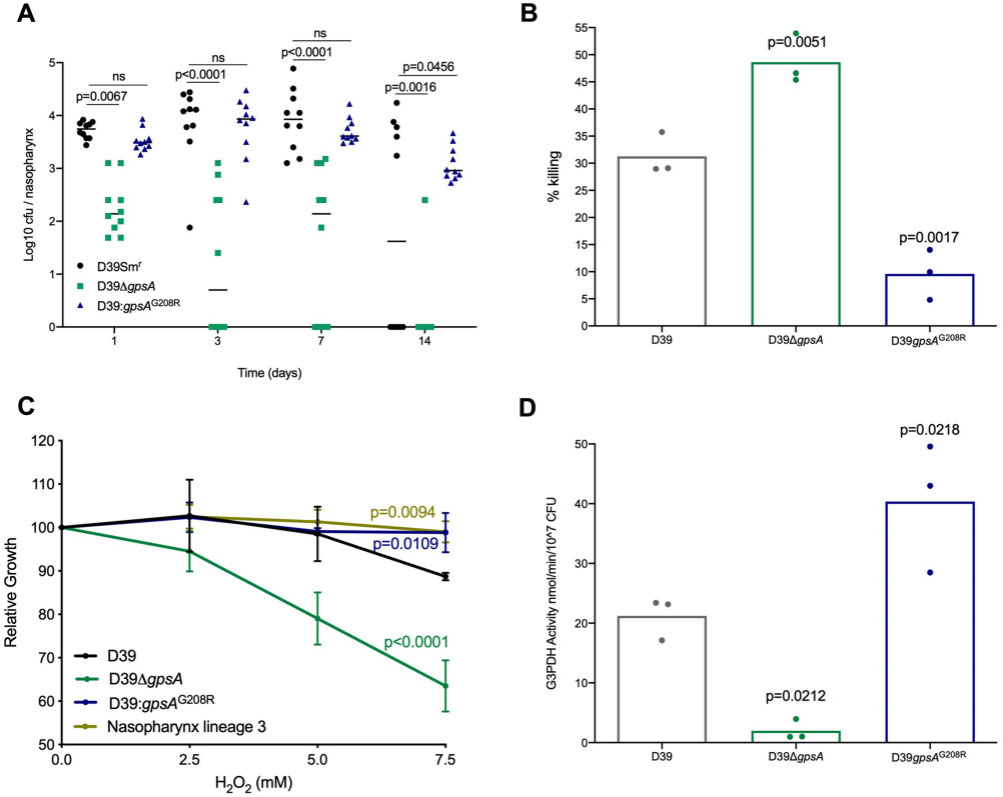
Glycerol-6-phosphate dehydrogenase is required for effective colonization of nasopharynx. (*A*) Mice were infected with a nasopharyngeal carriage-inducing dose of a streptomycin-resistant version of the ancestor D39 in which either a clean deletion of *gpsA* (D39Δ*gpsA*) or the SNP causing the G208R substitution (D39: *gpsA*^G208R^) had been generated. Control animals were infected with the streptomycin-resistant D39 ancestor expressing wild type *gpsA* (D39Sm^r^). Bacterial numbers were determined by serial dilution of nasopharynx homogenates onto selective agar at days 1, 3, 7, or 14 postinfection. *N* = 10 per group. P-values are from two-way ANOVA with Sidak’s multiple comparison test. (*B*) CFU counts following 30 min incubation in a 12 mM hydrogen peroxide (H_2_O_2_) solution. Data are presented as percentage killing vs the no H_2_O_2_ control for each strain. *P*-values are from a one-way ANOVA with Dunnett’s multiple comparison test vs D39. (*C*) Percentage reduction in growth (measured as area under the logistic curve [AUC]) for D39, D39*gpsA*, D39*gpsA*^G208R^, and nasopharynx lineage 3 in the presence of increasing concentrations of H_2_O_2_. *P*-values are from a two-way ANOVA with Dunnett’s multiple comparison test versus D39. Each point is the mean of three biological replicates, each containing three technical replicates. Error bars are standard deviation. (*D*) Glycerol-3-phosphate dehydrogenase (G3PDH) enzyme activity measured using a colorimetric assay. Activity was determined relative to a NADH standard curve and is presented as nmol NADH per minute per 10^7^ CFU. *P*-values are from a one-way ANOVA with Dunnett’s multiple comparison test versus the D39 ancestor. (*B*, *D*) Each data point represents a single biological replicate, each of which is the mean of three (*B*) or two (*D*) technical replicates.

G3PDH plays a role in membrane lipid metabolism and maintenance of intracellular redox potential (Lupien et al. 2013). We constructed a targeted deletion of *gpsA*, and individually reproduced the identified mutation that generated the G208R variant, in the ancestor D39 strain, using the sweet Janus system (Li et al. 2014). Before assessing the nasopharyngeal colonization potential of the resulting D39*gpsA* and D39*gpsA*^G208R^ strains, relative to a streptomycin resistant D39 control (D39Sm^r^), we first determined growth in nutrient broth (supplementary fig. 19, Supplementary Material online). Growth was comparable in D39Sm^r^, D39Δ*gpsA*, D39*gpsA*^G208R^, and nasopharynx-passaged lineages 3 and 4. The addition of 1 µg/ml gentamicin (the concentration used in agar during experimental passage) to broth led to a modest and comparable decrease in growth rates of all strains (supplementary fig. 19, Supplementary Material online). Furthermore, the gentamicin MIC50 was unaltered in D39*gpsA*^G208R^, relative to D39Sm^r^ (supplementary fig. 20, Supplementary Material online), suggesting that the SNP identified in nasopharynx-passaged lineages 3 and 4, conferring the G208R change, does not represent a gentamicin resistance mutation selected for during passage on agar. However, the gentamicin MIC50 of D39Δ*gpsA* was increased (supplementary fig. 20, Supplementary Material online), consistent with previous studies, in other species, that demonstrated associations of loss of function mutations in G3PDH-encoding genes with gentamicin resistance (Loss et al. 2019). Pneumolysin production was also unaltered by deletion or mutation of *gpsA* (supplementary fig. S21, Supplementary Material online). In vivo, D39Δ*gpsA* was markedly attenuated in its ability to colonize murine nasopharynx, showing reduced colonization density relative to D39Sm^r^ from day 1 postinfection and evidence of clearance as early as day 3 (fig. 7*A*). D39*gpsA*^G208R^, by contrast, showed prolonged maintenance of carriage, relative to D39Sm^r^, with 100% of mice still colonized at day 14 postinfection (fig. 7*A*).

It has been proposed that GpsA may be involved in the modulation of intracellular redox potential (Lupien et al. 2013), although the significance of this in the context of infection has not been explored. We sought to determine whether the G208R substitution in nasopharynx lineages 3 and 4 might impact resistance to reactive oxygen species (ROS). Pneumococci produce ROS as a byproduct of metabolism and are exposed to oxidative stress in the respiratory tract, both from co-colonizing microbes and from the actions of neutrophils (Yesilkaya et al. 2013). A hydrogen peroxide sensitivity assay revealed the significantly elevated susceptibility of D39Δ*gpsA* compared with the wild type, whereas D39*gpsA*^G208R^ was more resistant to H_2_O_2_ induced killing (fig. 7*B*). During long-term exposure to H_2_O_2_, both D39*gpsA*^G208R^ and a nasopharynx-passaged lineage, containing the same mutation, showed increased resistance, compared with both D39 and D39Δ*gpsA* (fig. 7*C*). Analysis of G3PDH activity confirmed a substantial reduction in D39*gpsA* and a significantly elevated level of activity in D39*gpsA*^G208R^, relative to D39 (fig. 7*D*). These data are consistent with the G208R variant of GpsA increasing the activity of G3PDH, and enhancing nasopharyngeal colonization potential of *S. pneumoniae* via increased resistance to oxidative stress.

## Discussion

Experimental evolution is a powerful tool for identification of genetic elements under selection within different environments (McDonald 2019). Cooper et al. (2020) recently showed the value of this approach for *Streptococcus pneumoniae* in mouse models, using a nasopharyngeal colonization model with a 19 F pneumococcal strain (BHN97x). There are some interesting commonalities in their findings and those reported here. Despite the bottlenecks occurring during experimental passage, both studies showed evidence of strong selection operating on pneumococcal populations. Parallelism and convergence were evident in independently evolving lineages within both studies and when comparing lineages derived from one study to those from the other, despite the different strain choice.

Cooper et al. highlighted the acquisition of frameshift mutations in *mutS* and demonstrated that loss of this gene conferred a hypermutator phenotype. We observed mutations in DNA repair genes *mutS*, *mutS2*, *mutX*, and *recN* with those in *mutX* reaching fixation in two lineages. The nonsynonymous mutations in *mutX* and *mutS2* were associated with increased mutation rates in niche-passaged pneumococci, consistent with a similar hypermutator phenotype.

Acquisition of mutations in genes encoding components of ABC transport systems was a further feature of both studies. We highlighted the *yknX* mutation in two nasopharynx lineages and one lung lineage, but also observed a mutation in *yknY* that reached fixation in nasopharynx lineage 9. Cooper and colleagues found a mutation in *yknZ* in one nasopharynx-passaged lineage. Collectively, YknXYZ form an ABC transport system involved in resistance to both antibiotics and host-derived antimicrobial peptides (Majchrzykiewicz et al. 2010; Yang et al. 2018). Further, Cooper et al. report mutations in *yheS_1, yheS_2* and *yheS_3*, whereas we identified a SNP within *yheH*. These genes also encode ABC transporter components, although the Yhe system is poorly characterized in pneumococcus. Nonsynonymous mutations in *yheS_2* were previously identified when comparing paired carriage and disease isolates of serotype 22 F (Cleary et al. 2016), suggesting that this gene may be involved in niche adaptation. Finally, both studies identified mutations in genes with roles in thiamine, copper, and pyruvate uptake or metabolism, highlighting the key requirement for trace metal scavenging and regulation of glycolysis during colonization (McAllister et al. 2004).

Although experiments were established with D39 stocks that originated from a single colony, we identified some potentially important genetic variation within the population used to found the 20 lineages. These variants likely arose from homologous recombination, coupled with a high mutation rate upon entry into stationary phase, during the overnight culture used to prepare the initial infection inoculum, as previously suggested by Cooper et al. (2020). Comparison of genetic ancestries of the lineages (supplementary figs. 7–16, Supplementary Material online) suggests that the subpopulations of the ancestor bearing the *D39N_01974* and *dnaK* polymorphisms were distinct, with each subsequently giving rise to separate genotypes. One of these often drove the other to extinction and, beyond passage 10, most lineages had lost either the *dnaK*-derived genotypes or those arising from the *D39N_01974* genotype. In lung lineages, the *dnaK* polymorphism became fixed in 8/10 lineages but was lost from the other two, where instead the *D39N_01974* polymorphism became fixed. In nasopharynx lineages, both genotypes were lost in 5/10 lineages. Examples of all three outcomes are shown in supplementary fig. 22, Supplementary Material online. It is possible that, within the nasopharynx, the *dnaK* or *D39N_01974* polymorphisms themselves did not significantly affect fitness and that the success of one or the other genotype came down to which was first to acquire a beneficial polymorphism, or to survive a population bottleneck. Intriguing, however, is the relative success of the *dnaK* polymorphism in lung lineages, where it became fixed in eight of ten lineages.

Although the lung is not the primary niche of pneumococcus, we opted to experimentally passage D39 through the lung environment, as colonization of this niche is associated with severe disease in humans. The nasopharynx and lungs differ in temperature, pH, microbiome composition, availability of nutrients, and degree of immune surveillance (Walsh and Camilli 2011; Man et al. 2017; Aprianto et al. 2018). The challenges posed to pneumococci by cocolonizing microbes differ significantly between the two niches, with the nasal microbiome heavily influenced by skin commensals (Rasmussen et al. 2000; Human Microbiome Project 2012), whereas the lung microbiome is thought to be more closely related to that of the oral cavity (Bassis et al. 2015). Such factors may have influenced the evolutionary trajectories observed in this study.

The lung passage process might theoretically have led to the emergence of variants with increased virulence, able to better utilize environmental resources, and overwhelm the host clearance mechanisms. Alternatively, those able to persist longer by virtue of inducing more muted inflammatory responses might have been selected. There are potential examples of both outcomes amongst the ten lung lineages. Lineage 6 produced significantly more pneumolysin than the ancestor, and showed comparable virulence, although it was cleared less efficiently by host defence mechanisms. Lineages 1, 2, 4, 8, and 10, by contrast, produced significantly less pneumolysin than the ancestor and all except lineage 10 showed decreased virulence but increased persistence. Thus, persistence achieved by inducing less severe disease appears the more common route to success for pneumococci in this experimental system. This may be a consequence of the process by which the populations were transferred between hosts. In the absence of a need to stimulate the inflammation normally required to achieve transmission, downregulation of pneumolysin may be advantageous, although the molecular mechanisms underpinning this are unclear, in the absence of variants in *ply* or flanking regulatory regions.

Pneumococcal lineages differ in their abilities to cause pneumonia and invasive pneumococcal disease (Sleeman et al. 2006), consistent with some being better adapted to thrive in the lung environment than others. By bypassing the carriage step that would ordinarily precede lung infection, we hoped to select for traits specifically advantageous in the lung environment. We observed only nine variants shared by nasopharynx and lung lineages but that were not present in the control passage or ancestor, as compared with 245 variants unique to nasopharynx lineages and 118 unique to lung. Although only a small fraction of identified variants could be considered adaptive mutations under positive selection, it was notable that the examples of potential parallelism or convergence we identified tended to be niche-specific. Fixed polymorphisms in *gpsA* and *pvaA* were only observed in nasopharynx lineages, whereas those in *mutX1*, *lafA*, and *sdhB* were unique to lung lineages. Only *yknX* showed evidence of selection in both niches, with a SNP causing a leucine to valine change at position 333 becoming fixed in one lung lineage, and a SNP causing a serine to asparagine substitution at position 331 becoming fixed in two nasopharynx lineages. In the nasopharyngeal carriage model that was used to experimentally passage pneumococci, occasional dissemination or seeding of pneumococci to the lower respiratory tract was observed (supplementary table S3, Supplementary Material online). Low-density colonization of nasopharynx occurs in the lung infection model, but bacterial numbers are only 1–2% of those seen in lung. Lung-colonized bacteria were not transferred during passage of the nasopharynx lineages, nor were nasopharynx-colonized bacteria transferred during passage of the lung lineages. However, movement of bacteria between the two niches might have contributed to evolutionary trajectories. Any such effect was likely minor, given the low densities and frequencies of seeding observed, but must be considered. A further limitation when comparing nasopharynx- and lung-passaged lineages is that the nasopharynx lineages spent ∼5-fold longer within their in vivo niche. Confirmation that identified variants are truly niche-specific will require mechanistic analysis of the type performed in this study for *gpsA*, by reproducing mutations individually or in combination and testing effects on nasopharynx and lung colonization.

Population size is a key determinant of the likelihood of observing parallel evolutionary change (Bailey et al. 2017) but the relationship can be confounded by spatial effects in nonhomogenous populations, such as those colonizing host surfaces. Studies in other systems have demonstrated the influence of a microbe’s position within a population on selection and evolution (Kim et al. 2014; Baym et al. 2016). Determining the population sizes in the infection models used here is not possible with the available data, but some indication can be gleaned from the colony counts recovered from nasopharynx and lungs between each passage. Infection of lungs was established with a dose of 10^6^ colony-forming units (CFU), whereas nasopharyngeal carriage was established with 10^5^ CFU. We recovered a median of 4.65 × 10^6^ CFU from lungs in the lung infection model, and 3.70 × 10^4^ CFU from nasopharynx in the nasopharyngeal carriage model. These figures suggest some bottleneck effects during the establishment of nasopharyngeal infection. Median recovery changed little with increasing passage number in the lung model (4.55 × 10^6^ at passage 1 vs. 5.05 × 10^6^ at passage 20) but increased ∼5-fold in the nasopharynx model (1.30 × 10^4^ at passage 1 vs. 6.99 × 10^4^ at passage 20). Further bottlenecks are likely introduced, in both models, by host immune and inflammatory responses to infection. The dose of bacteria administered during lung passage induces robust innate immunity and at least partial clearance of infection (Kadioglu et al. 2000), whereas the lower dose and volume used to establish nasopharyngeal infection causes more muted host responses (Neill et al. 2014) and, presumably, less tight bottlenecks. Regardless of bottleneck effects, the observation of parallel evolution at locus and nucleotide level in both infection models is suggestive of adaptive evolution, rather than population changes attributable to genetic drift (Orr 2005).

The same nonsynonymous SNP in *gpsA* was identified in two nasopharynx lineages and a recent study of pneumococcal carriage in infants in the Gambia also identified a high frequency of mutation in this gene (Chaguza et al. 2020) (table 3). Deletion of *gpsA* in the ancestor strain compromised nasopharyngeal carriage, whereas the identified SNP conferred increased carriage potential and resistance to oxidative stress, when reproduced individually in D39. Glycerol 3-phosphate dehydrogenase reduces NADP^+^ to NADPH during the conversion of sn-glycerol 3-phopshate to glycerine phosphate, thereby helping maintain intracellular redox potential. Increased redox control during carriage may be beneficial in the context of competition with co-colonizing microbes or in resistance to neutrophil responses. Polymorphisms conferring such phenotypes may have consequences for antimicrobial resistance in pneumococcus, as antibiotics including penicillin and ciprofloxacin induce production of ROS (Fani et al. 2011; Lupien et al. 2013).

We identified increased adhesion to airway epithelial cells in three of ten nasopharynx-passaged lineages and reduced pneumolysin production in nine of ten lineages. Both these traits could compromise transmission while promoting stable carriage. An assessment of transmission potential in niche-adapted lineages will be an important next step for this work. If nasopharynx-passaged lineages have increased potential for noninvasive carriage together with loss of transmission potential, then they may be suitable starting points for the development of live-attenuated pneumococcal vaccines, although requiring further laboratory attenuation to prevent reversion to virulence or acquisition of new traits in vivo via recombination.

An open question is an extent to which the genetic changes we observed might be constrained in a natural infection setting. In our model, the process of transmission—associated with tight bottlenecks (Kono et al. 2016)—was bypassed by manual transfer of pneumococci between animals. The need to balance attachment and colonization with shedding and transmission is likely a major determining factor in the trajectory of pneumococcal evolution and the trade-off between transmissibility and within-host fitness has been recently highlighted (Jacobs and Weiser 2020). The authors outline the role played by competitive exclusion in restricting population diversity, whereby the first variant to colonize prevents later arrivals from doing so. The degree to which this phenomenon might impact evolution in our experimental system, which utilized manual administration of the bacterial population as opposed to natural exposure, is unclear. We did observe loss of some genotypes between passages but also instances where multiple, co-existing genotypes were maintained for several passages.

One important caveat to the findings presented relates to the anatomical, biochemical, microbiological, and immunological differences between the mouse and human respiratory tracts. In addition to adapting pneumococci to the nasopharynx or to the lungs, the passage process we describe might select for variants that give an advantage specific to colonization of murine respiratory tissue. We are, however, encouraged by the comparison between our study and that of Chaguza et al. (2020), demonstrating that 43% of genes in which we observed fixed nonsynonymous SNPs, and 39% of the total number of genes in which we observed variants, were also frequently selected targets of mutation in human colonization.

Collectively, our findings suggest that in vivo experimental evolution can be a valuable tool for the study of host–pathogen interactions and disease mechanisms. Environmental differences between the niches of the upper and lower airway may be sufficient to alter the trajectory of evolution in populations of bacteria exposed to one or the other. We identified several examples of potential parallel evolution in independently passaged populations and the genes under selection highlight the importance of cell surface modification and metabolic versatility in pneumococcal colonization and disease processes. In vivo experimental evolution approaches can be used to identify pathogen genes playing key roles in within-host processes. This was exemplified by *gpsA*, that appeared to be under strong selection in nasopharynx, and in which we identified a single nucleotide change conferring phenotypic change and increased within-host fitness.

## Materials and Methods

### Media and Growth Conditions

*Streptococcus pneumoniae* D39 (serotype 2, NCTC 7466) was grown at 37 °C, 5% CO_2_ on blood agar base (BAB) (Oxoid) supplemented with 5% (vol/vol) defibrinated horse blood (Oxoid) and 1 µg/ml gentamicin. Overnight cultures were prepared in 10 ml of brain heart infusion (BHI) broth (Sigma). Bacteria were identified as pneumococci by α-hemolysis on blood agar and by optochin sensitivity. D39 used in this study was generously provided by Prof Tim Mitchell (University of Birmingham).

### Ethics Statement

All mouse infection work was performed at the University of Liverpool with prior approval by the UK Home Office (project licence PB6DE83DA) and the University of Liverpool Ethics Committee. For all experiments, 6–8-week-old female CD1 mice were purchased from Charles River (Margate, United Kingdom). Mice were randomly assigned to a cage of 1–5 mice, depending on the experiment, on arrival at the unit, by staff with no role in study design. Mice were housed in individually ventilated cages and allowed to acclimatize for 7 days prior to infection.

### Experimental Evolution in Mouse Infection Models

A standardized suspension of the D39 ancestor strain was prepared for inoculation by overnight growth in BHI from a single colony. The next day, subculture was performed and the bacteria allowed to grow to mid-exponential phase (∼6 h) before storage at −80 °C. For the initial infection, this suspension was thawed at room temperature and bacteria were harvested by centrifugation and suspended in phosphate-buffered saline (PBS). For the carriage model, ten mice were intranasally infected under light anaesthesia, using a mix of oxygen and isoflurane, with 1× 10^5^ CFUs in 10 µl saline. To induce pneumonia, ten mice were infected intranasally with 1.5 × 10^6^ CFU, in 50 µl saline, to ensure that the pneumococcal population was deposited directly into the lung. The 20 infected mice were treated as founders of 20 independent pneumococcal lineages, ten of which were passaged through nasopharynx and ten which were passaged through lung. In the carriage model, the bacteria remained in the nasopharynx for 7 days for each passage and, for the pneumonia model, bacteria were in the lung environment for 48 h, or less if the mice reached the severity endpoint before this time. The time the lineages spent in the lungs ranged from 22 to 48 h per passage, with an average time of 31.91 h. After each passage, pneumococci were recovered from the nasopharynx, in the carriage model, or lungs, in the pneumonia model. This was achieved by dissection of the organ of interest, followed by disruption of tissue in saline using a hand-held tissue homogeniser and then plating of homogenates onto gentamicin BAB agar. After this minimal passage on agar, the entire population of recovered pneumococci for each lineage was resuspended into 1 ml of BHI/10% glycerol, which was split 500:250:250 between three tubes and stored at −80 °C for future use in infection experiments, phenotypic analysis or genome sequencing. One of the 250-µl frozen aliquot tubes was used to determine the CFU by Miles and Misra and to re-infect a new mouse for the next round of passage. This passage process was repeated a total of 20 times for each of the separately evolving lineages in the carriage and pneumonia models of infection. For all subsequent in vitro and in vivo phenotypic experiments performed with the 20× passaged lineages, a dilution of the total population was prepared from the BHI/10% glycerol stocks, without further culture. Figure 1 gives an overview of the in vivo experimental evolution process undertaken during this study.

### Control Laboratory Passage on BAB Agar

The same D39 ancestor stock used for the in vivo evolution experiments was used to streak ten gentamicin BAB plates with an optochin disc and incubated for 20–24 h at 37 °C in 5%. For the next passage for each lineage, the entire population was collected into PBS or BHI/10% glycerol (for long-term storage of passage numbers 1, 5, 10, 15, and 20) before a dilution of the collected population equivalent to ∼10^5^ CFUs was plated onto a fresh gentamicin BAB plate. This process was repeated 20 times for the ten separately evolving lineages. Illumina sequencing was performed on passage 20 populations. In the final analysis, only eight of the ten lineages were included due to contamination of the DNA used for sequencing.

### Illumina Sequencing and Read Processing

DNA extraction protocols are detailed in supplementary methods, Supplementary Material online. Short read sequencing was performed to produce 151-bp paired-end reads on an Illumina HiSeq2500 at the Wellcome Trust Sanger Institute, United Kingdom. This was carried out for the ancestor and for passages 1, 5, 10, 15, and 20, for both nasopharynx and lung evolved lineages. Short read sequencing was also carried out for the passage 20 control lineages. To obtain a deep sequence coverage of the ancestor D39 strain, DNA was paired-end sequenced on an Illumina NextSeq 500 system using 150-bp read lengths, by Vertis Biotechnologie AG, Germany. Analysis of sequencing data was carried out using a virtual machine hosted by the Cloud Infrastructure for Microbial Bioinformatics (CLIMB) consortium (Connor et al. 2016). Raw Illumina reads were processed using Trim Galore (v0.4.4) with Cutadapt (v1.9.1) for paired-end reads, using default settings, to remove short low-quality reads (<20 bp), trim Illumina adaptor sequences, and eliminate poor-quality bases from the sequences (<Q20). FastQC (v0.11.5) confirmed that trimmed reads were of sufficient quality for analysis. Trimmed FastQ files of Illumina paired end sequence reads are available from NCBI (Bioproject Accession Number: PRJNA658145).

### PacBio Long Read Sequencing and Genome Assembly

Single molecule real-time (SMRT) sequencing (Pacific Biosciences, PacBio, CA) of the ancestor D39 isolate (available from NCBI under BioProject accession number PRJNA658145) was performed at the Wellcome Trust Sanger Institute (Cambridge, United Kingdom). The resulting BAM file was converted to FASTQ with bamToFastq (v2.28.0). PacBio long reads were filtered by quality using Filtlong (v0.2.0- https://github.com/rrwick/Filtlong) and assembled with the Flye Assembler (v2.4.2) (Kolmogorov et al. 2019); default settings were applied for both programmes. The resulting contig, with 469× coverage depth, was polished using the PacBio SMRT minimap2 tool (v2.15-r905) and Racon Polisher (v1.3.3 - https://github.com/isovic/racon), with default settings applied. A consensus sequence of the ancestor was generated using PacBio SMRT tools Blasr alignment (v5.1) and Arrow (v2.3.3). This was then corrected by aligning the sequence with the ancestor Illumina reads using bwa mem (v0.7.17-r1188) and re-assembling with Pilon (v1.22) (Walker et al. 2014). The resulting corrected consensus sequence was annotated using Prokka (v1.13) (Seemann 2014) and run through the Quality Assessment Tool for Genome Assemblies (QUAST, v4.6.3) to obtain the assembly statistics. Comparison of Prokka annotation with the recent D39 genome assembly of Slager et al. (2018) is shown in supplementary data set 1, Supplementary Material online.

### Variant Calling

Variants were detected from processed Illumina reads for the nasopharynx and lung evolved lineages at passages 1, 5, 10, 15, and 20, as well as the control lineages at passage 20. The short sequence reads were mapped to the D39 ancestor corrected consensus sequence to identify mutations, using the Breseq programme (v0.31.1) (Barrick et al. 2014). Default settings were applied with the predict-polymorphisms function. This process identified the percentage frequency of variants within a lineage population at each passage. Synonymous and nonsynonymous SNPs as well as insertions/deletions and intergenic mutations were detected. The deeper sequenced lllumina reads for the ancestor strain (×300 coverage) were also used to detect variants within the original D39 ancestor inoculum, with the polymorphism-frequency-cut off parameter set to >2%.

### Comparison with Clinical Isolate Data

We analysed pneumococcal genomes from a recently published data set of isolates sampled longitudinally in newborn infants, to assess whether the mutations identified during experimental evolution occur frequently within hosts during natural carriage episodes (Chaguza et al. 2020). We mapped the reads of each isolate against the ancestral D39 reference genome and generated a consensus alignment for each episode using Snippy (v4.3.6) (https://github.com/tseemann/snippy). The genomic positions with SNPs during each carriage episode were identified, annotated, and compared between episodes to identify episodes with changes occurring in parallel at the same location using BioPython (Cock et al. 2009).

### Pneumonia and Carriage Experiments in Mice

Mice were infected with standardized pneumococcal isolate stocks at a dose of 1 × 10^6^ CFU in 50 µl saline via intranasal administration under light anaesthesia, to induce acute pneumococcal disease, or with 1 × 10^5^ CFU in 10 µl saline to induce asymptomatic nasopharyngeal colonization. Mice were monitored for disease signs and pain score was determined using the scheme of Morton (Morton 1985). For survival experiments, mice were monitored for 7 days and culled once lethargic. For time point experiments, mice were culled at 0, 12, 24, and 36 h postinfection for pneumonia experiments, or at 1, 3, 7, 14, and 21 days postinfection for carriage experiments. Nasopharynx, lungs, and blood samples were taken and pneumococcal CFU determined via Miles and Misra dilution onto gentamicin BAB plates.

### Analysing Pneumococcal Growth Dynamics in the Presence of Hydrogen Peroxide

Growth assessment was performed as detailed in supplementary methods, Supplementary Material online, with the following modifications. Pneumococcal isolates were grown in triplicate in BHI in 96-well plates in the presence of hydrogen peroxide (H_2_O_2_, Sigma) at concentrations 0, 2.5, 5.0, and 7.5 mM. Growth dynamics were analysed with the R package GrowthCurver (Sprouffske and Wagner 2016) (version 0.3.0) in R-Studio (v3.6.1) and the percentage reduction in the AUC values for each isolate in the presence of H_2_O_2_ was calculated in comparison to 0 mM.

### Hydrogen Peroxide Sensitivity Assays

Mid-log phase pneumococci were added in duplicate to microtiter plate wells and incubated at 37 °C for 30 min in BHI with 12 mM of H_2_O_2_. The CFU/mL of each isolate was determined via Miles and Misra dilution onto gentamicin BAB plates and the percentage killing was calculated for each test isolate in comparison to the untreated (BHI only) control.

### Glycerol-3-Phosphate Dehydrogenase Assay

G3PDH activity was measured using a colorimetric assay kit (Abcam). Overnight cultures of isolates were centrifuged for 15 min at 3,000 rpm and the pellet resuspended in 200 ul of G3PDH assay buffer and lysed with the addition of sodium deoxycholate at 10%. This was incubated at room temperature for 10 min, centrifuged for 15 min at 3,000 rpm and the supernatant used for the assay following the manufacturer’s instructions. Absorbance readings at 450 nm were taken at time zero and following a 60-min incubation at 37 °C and the activity of G3PDH for each isolate was calculated by comparison to a NADH standard curve.

## Statistical Analysis

GraphPad Prism version 8.2.1 was used for statistical analysis. Statistical tests undertaken for individual experiments are detailed in the respective figure legends. *P* < 0.05 was considered to be statistically significant. Data were tested for normality and to define the variance of each group tested. All multiparameter analyses included corrections for multiple comparisons and data are presented as mean ±  SD unless otherwise stated. Additional methodological information can be found in supplementary methods, Supplementary Material online.

## Supplementary Material

Supplementary data are available at *Molecular Biology and Evolution* online.

## Supplementary Material

msab018_Supplementary_Data
