# Supplementary material for: Pneumococcal Colonization and Virulence Factors Identified Via Experimental Evolution in Infection Models
Source: Mol Biol Evol. 2021 Jan 27;38(6):2209–26. doi: 10.1093/molbev/msab018 (PMC8136498; doi:10.1093/molbev/msab018)
Supplement: msab018_Supplementary_Data [file msab018_supplementary_data.zip › Green_et_al_SuppFigures_04122020.pdf]

Supplementary Figures

Pneumococcal colonisation and virulence factors identified via experimental evolution in infection models

Angharad E Green et al.

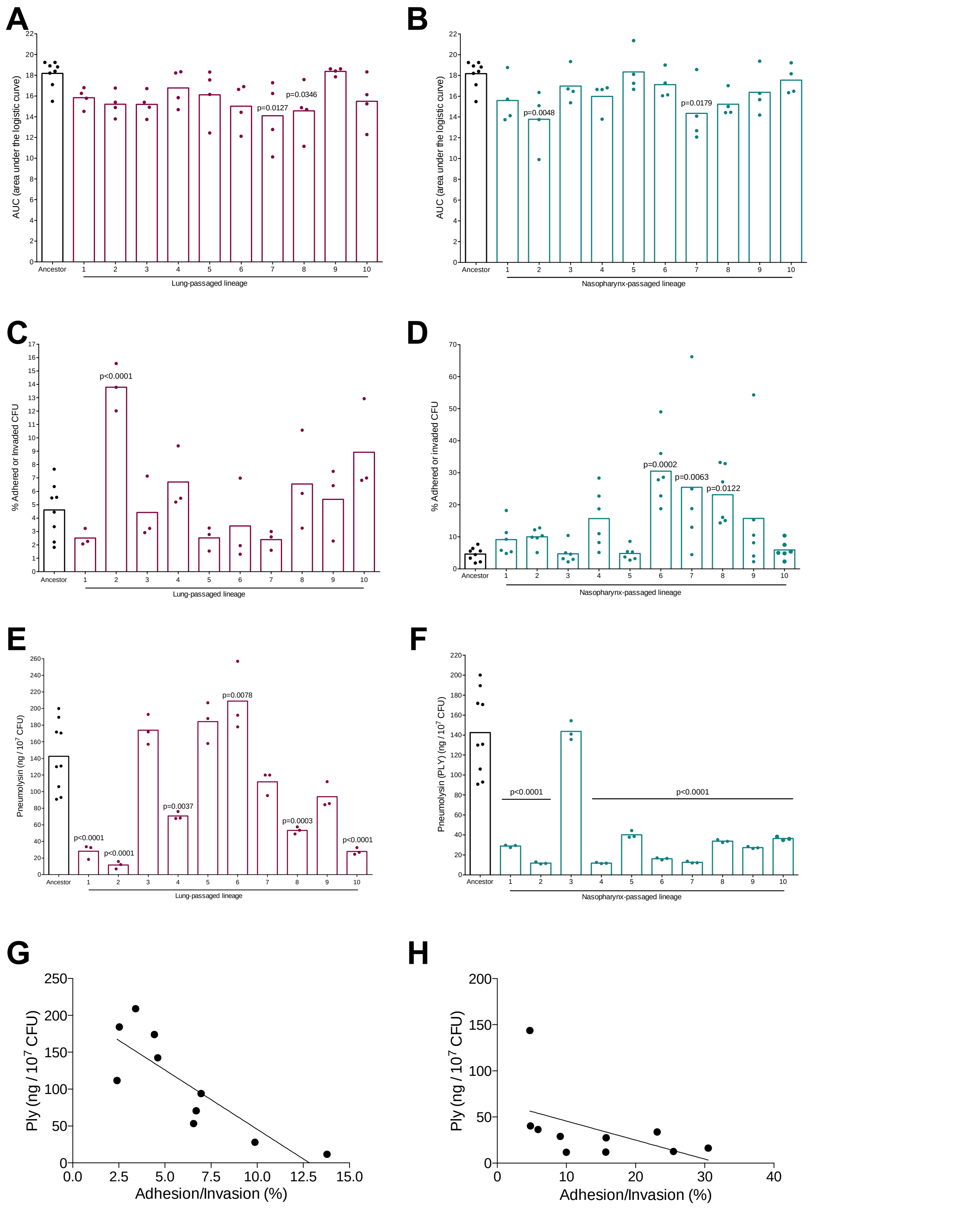

Supplementary Figure 1. Phenotypes of niche-passaged pneumococci. Twenty times passaged pneumococcal lineages from lung or nasopharynx were compared to the ancestral D39 population. Growth over 24 hours in nutrient broth is presented as area under the curve (AUC) values for **(A)** lung-passaged and **(B)** nasopharynx-passaged lineages. **(C, D)** Adhesion efficiency to A549 airway epithelial cells was determined following a 1 hour co-incubation. **(E, F)** Pneumolysin concentrations in lysates prepared from $1x10^7$ mid-log phase pneumococci were determined by ELISA and comparison to a recombinant pneumolysin standard curve. In **(A-F)** p-values were determined by one-way ANOVA with Dunnett's multiple comparison test vs the ancestor. Data points show individual biological replicates, each of which is the mean of three technical replicates. For each lung **(G)** and nasopharynx **(H)** passaged lineage, mean adhesion percentage was plotted against mean pneumolysin concentration and a line of best fit determined by linear regression.

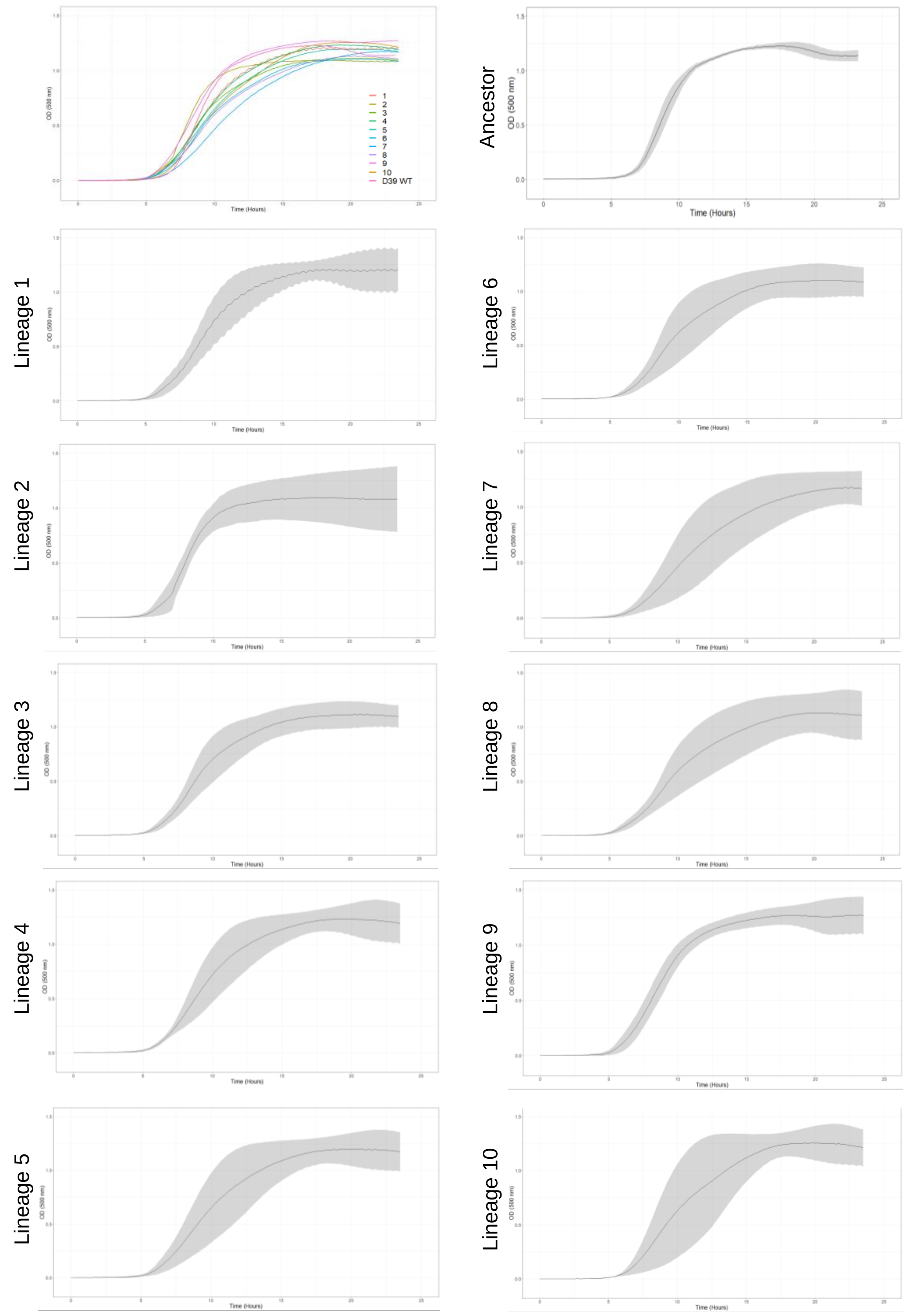

Supplementary Figure 2. Growth of lung-passaged lineages in laboratory media. Growth over 24 hours in nutrient broth. Line shows mean optical density at 500 nm, measured every 15 minutes. Shaded area represents standard deviation. Data shown are a composite of the same biological replicates used to generate AUC data in Supplementary Figure 1A.

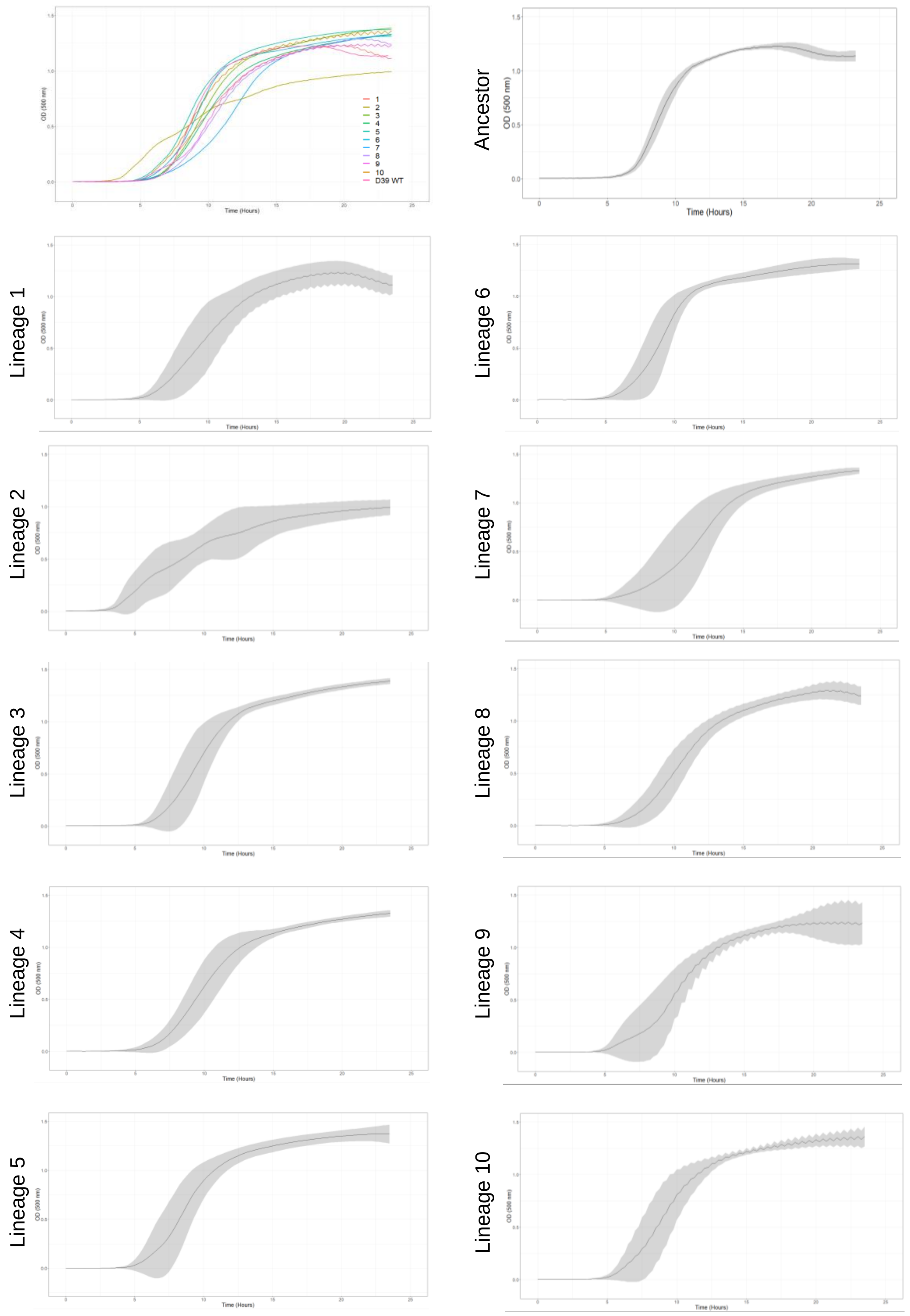

Supplementary Figure 3. Growth of nasopharynx-passaged lineages in laboratory media. Growth over 24 hours in nutrient broth. Line shows mean optical density at 500 nm, measured every 15 minutes. Shaded area represents standard deviation. Data shown are a composite of the same biological replicates used to generate AUC data in Supplementary Figure 1B.

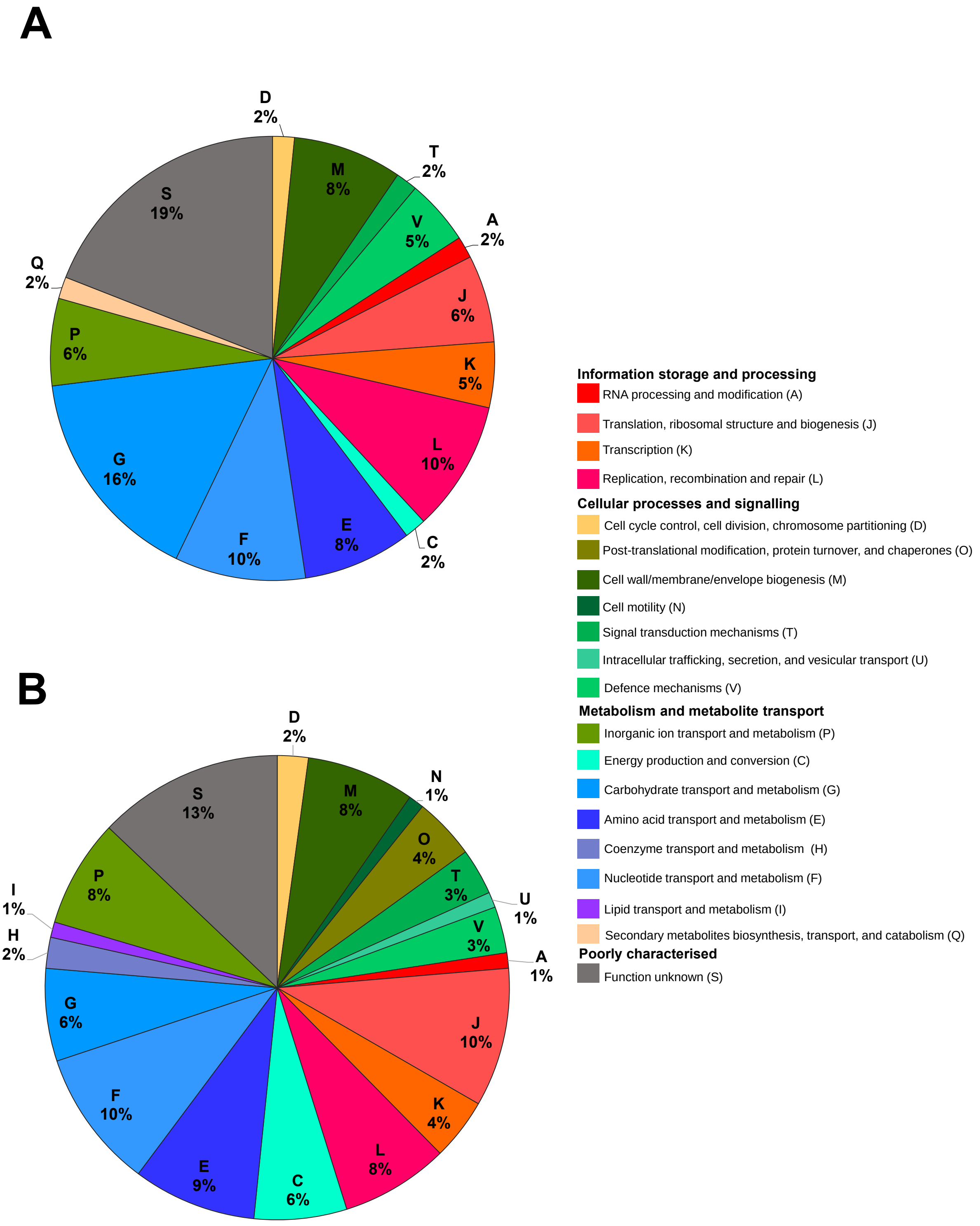

Supplementary Figure 4. COG analysis of passage 20 variants. Analysis of variants in (A) lung-passaged, and (B) nasopharynx-passaged lineages. COG categories were assigned to genes for all mutations found in the lung and nasopharynx passage 20 isolates. The percentage of gene variants mapping to the various COG functional groups is represented as pie charts. The COG categories for those genes with mutations appearing in more than one lineage are represented only once and gene variants also found in the control passage were removed from this analysis. The segments of the pie charts are coloured according to the COG-assigned function, as depicted in the key.

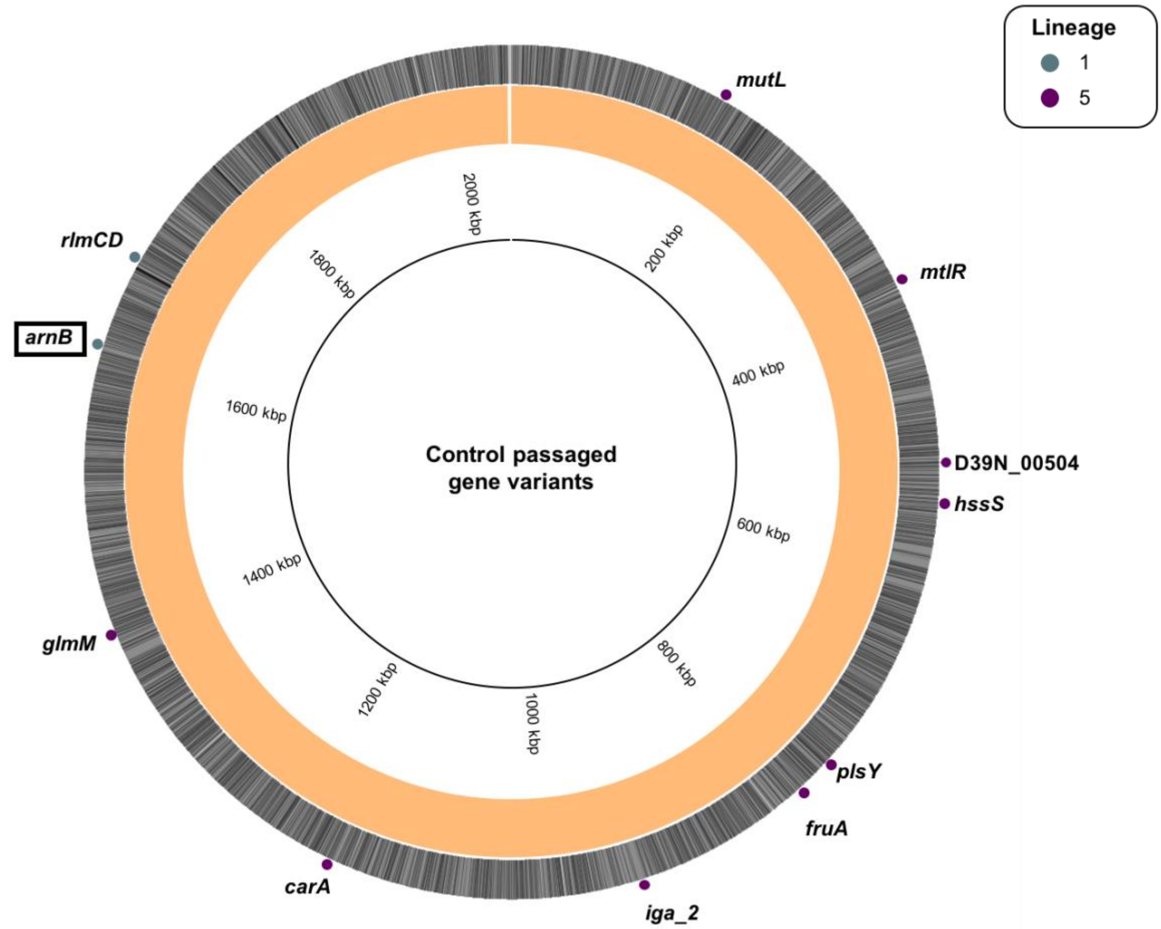

Supplementary Figure 5. Fixed non-synonymous single nucleotide polymorphisms in pneumococci passaged on gentamicin blood agar. Genome maps of the D39 ancestor assembly, highlighting the non-synonymous single nucleotide polymorphisms identified in pneumococci passaged twenty-times on blood agar containing gentamicin. Labels show the name and position of the gene in which the SNP occurs, with the circles coloured according to the lineage where the variant was identified. Of the eight control passage lineages sequenced, only lineages 1 and 5 contained fixed non-synonymous mutations. The box around *arnB* denotes that the same mutation was identified in *in vivo* passaged pneumococci.

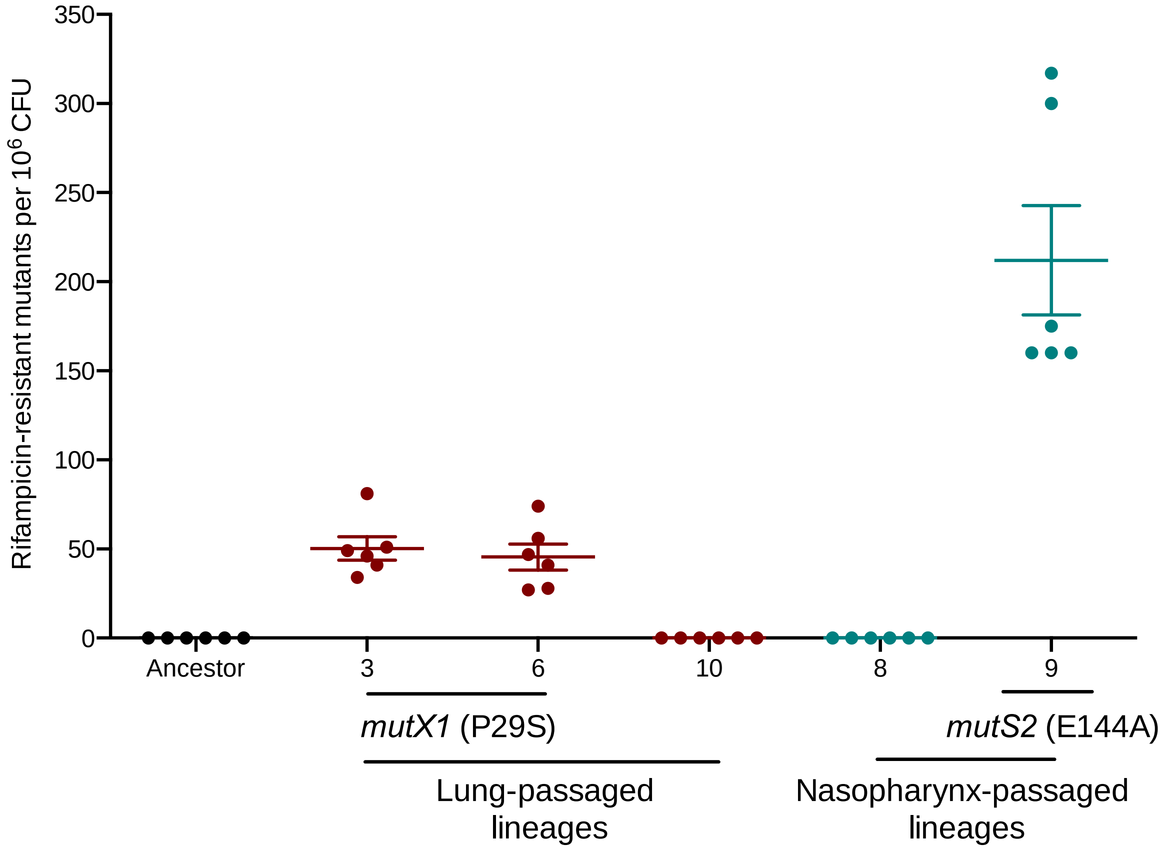

Supplementary Figure 6. Spontaneous rifampicin resistance mutants in niche passaged lineages with mutations in DNA repair genes. The number of spontaneous rifampicin resistant mutants detected per 10^6 plated pneumococci on blood agar containing 0.125 µg/ml rifampicin. The genes in which non-synonymous mutations were acquired are shown, with the resulting amino acid change shown in brackets. Lung-passaged lineage 10 and nasopharynx-passaged lineage 8 were included as additional controls, as neither lineage acquired mutations in DNA repair genes during experimental passage.

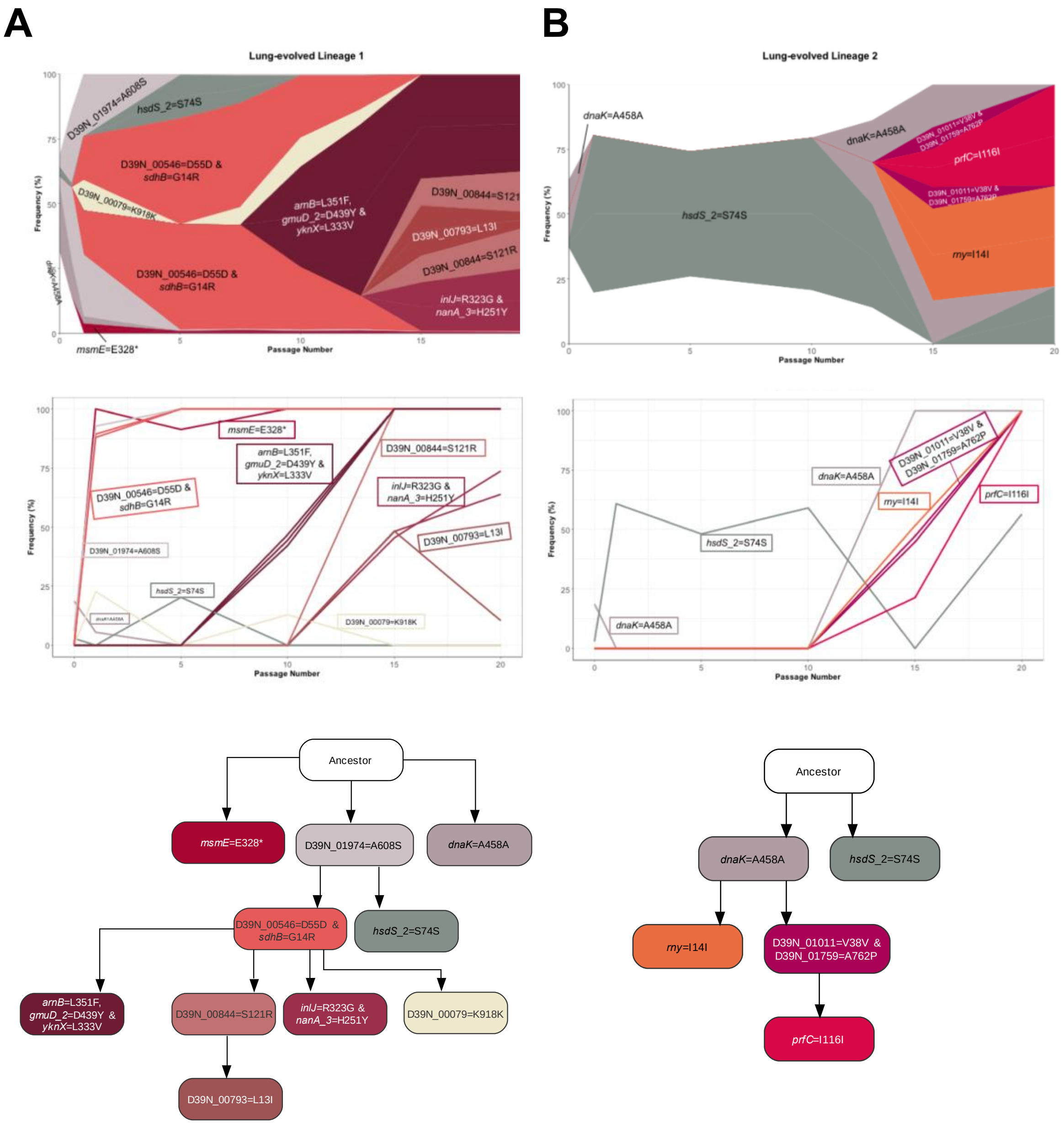

Supplementary Figure 7. Genotype emergence and dynamics in lung passaged pneumococcal populations. (A) Lung-passaged lineage 1. **(B)** Lung-passaged lineage 2. Panels, from top-to-bottom, are Muller plots, genotype frequency plots and genotype ancestry trees.

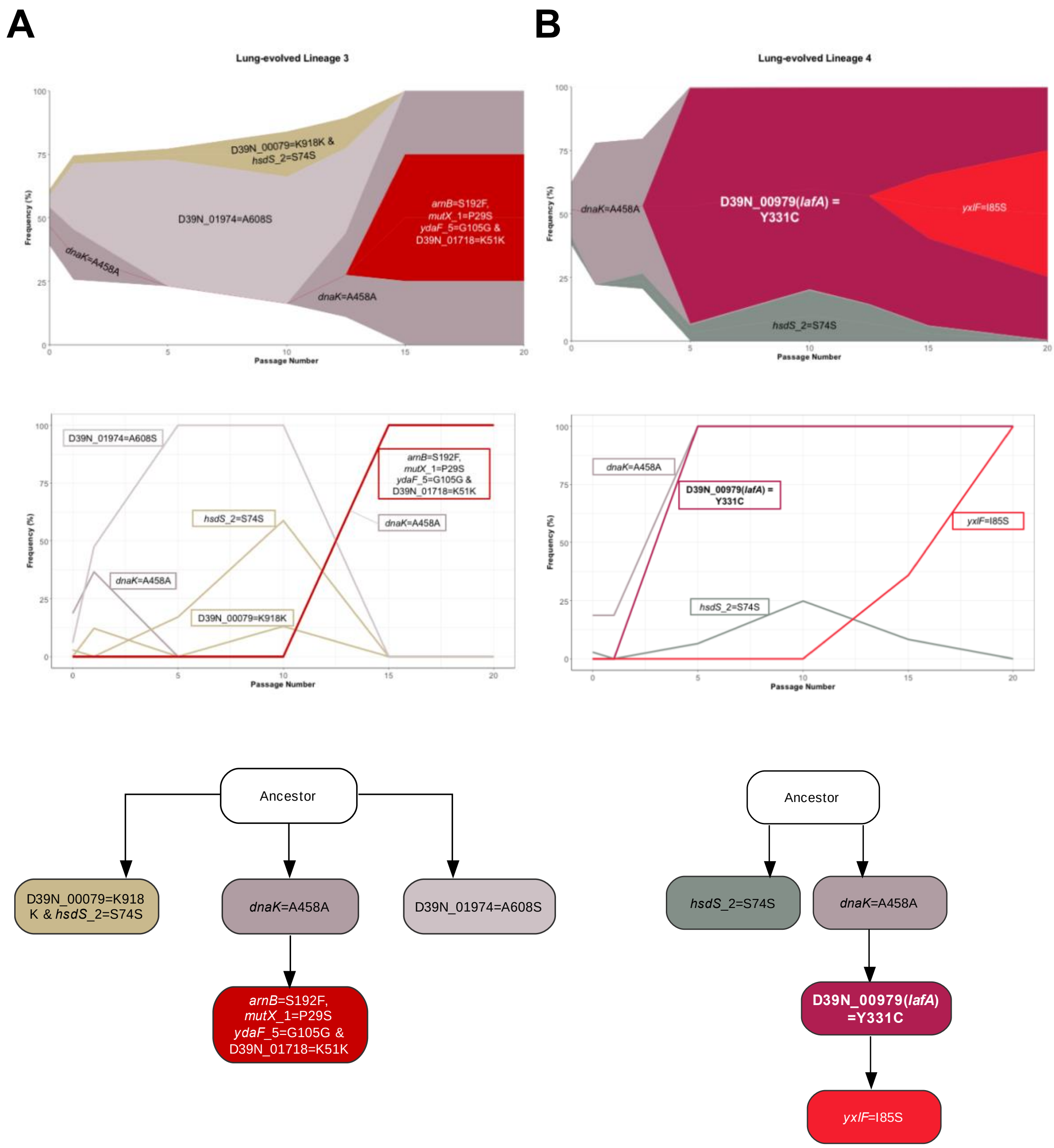

Supplementary Figure 8. Genotype emergence and dynamics in lung passaged pneumococcal populations. (A) Lung-passaged lineage 3. **(B)** Lung-passaged lineage 4. Panels, from top-to-bottom, are Muller plots, genotype frequency plots and genotype ancestry trees.

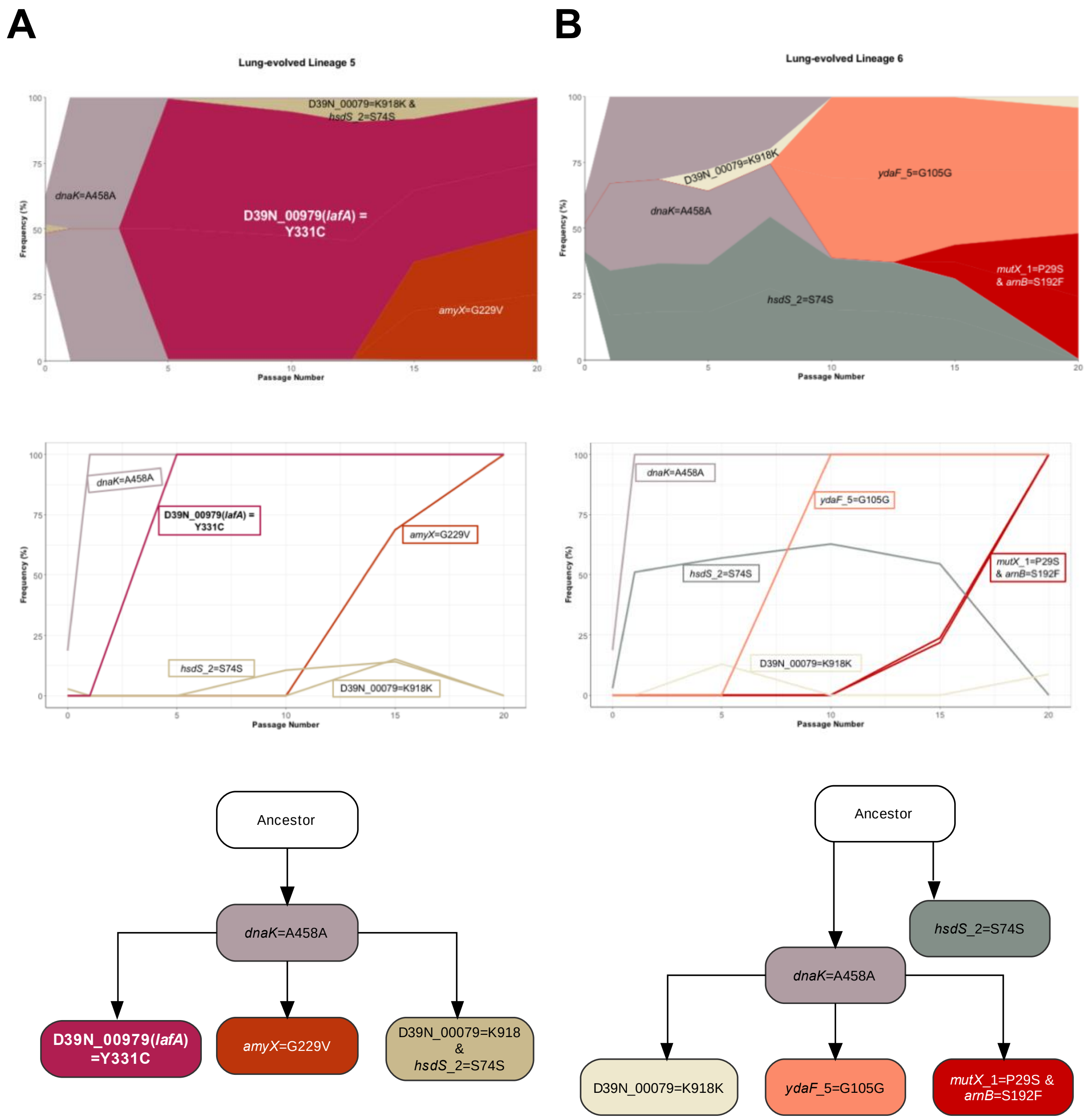

Supplementary Figure 9. Genotype emergence and dynamics in lung passaged pneumococcal populations. (A) Lung-passaged lineage 5. **(B)** Lung-passaged lineage 6. Panels, from top-to-bottom, are Muller plots, genotype frequency plots and genotype ancestry trees.

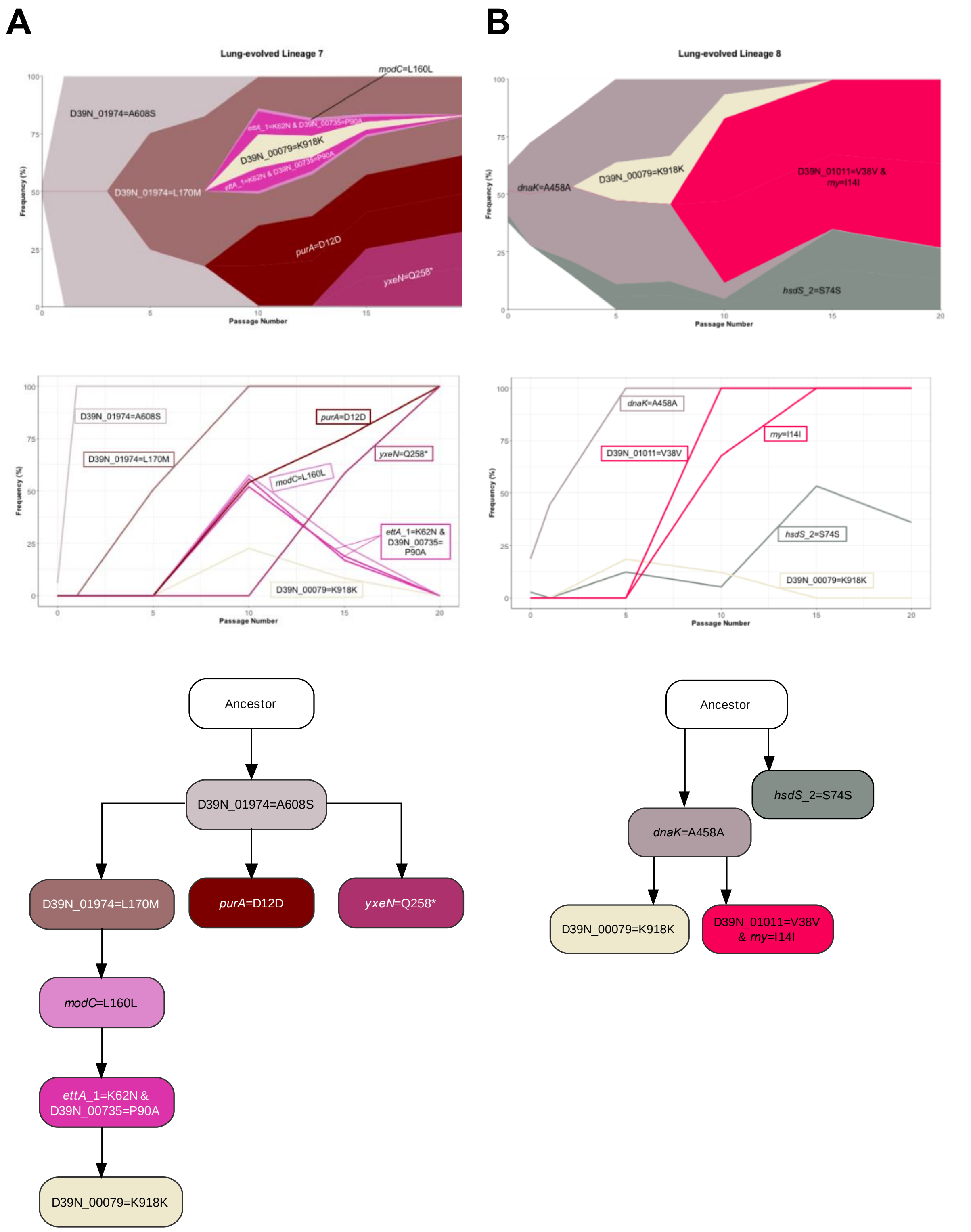

Supplementary Figure 10. Genotype emergence and dynamics in lung passaged pneumococcal populations. (A) Lung-passaged lineage 7. **(B)** Lung-passaged lineage 8. Panels, from top-to-bottom, are Muller plots, genotype frequency plots and genotype ancestry trees.

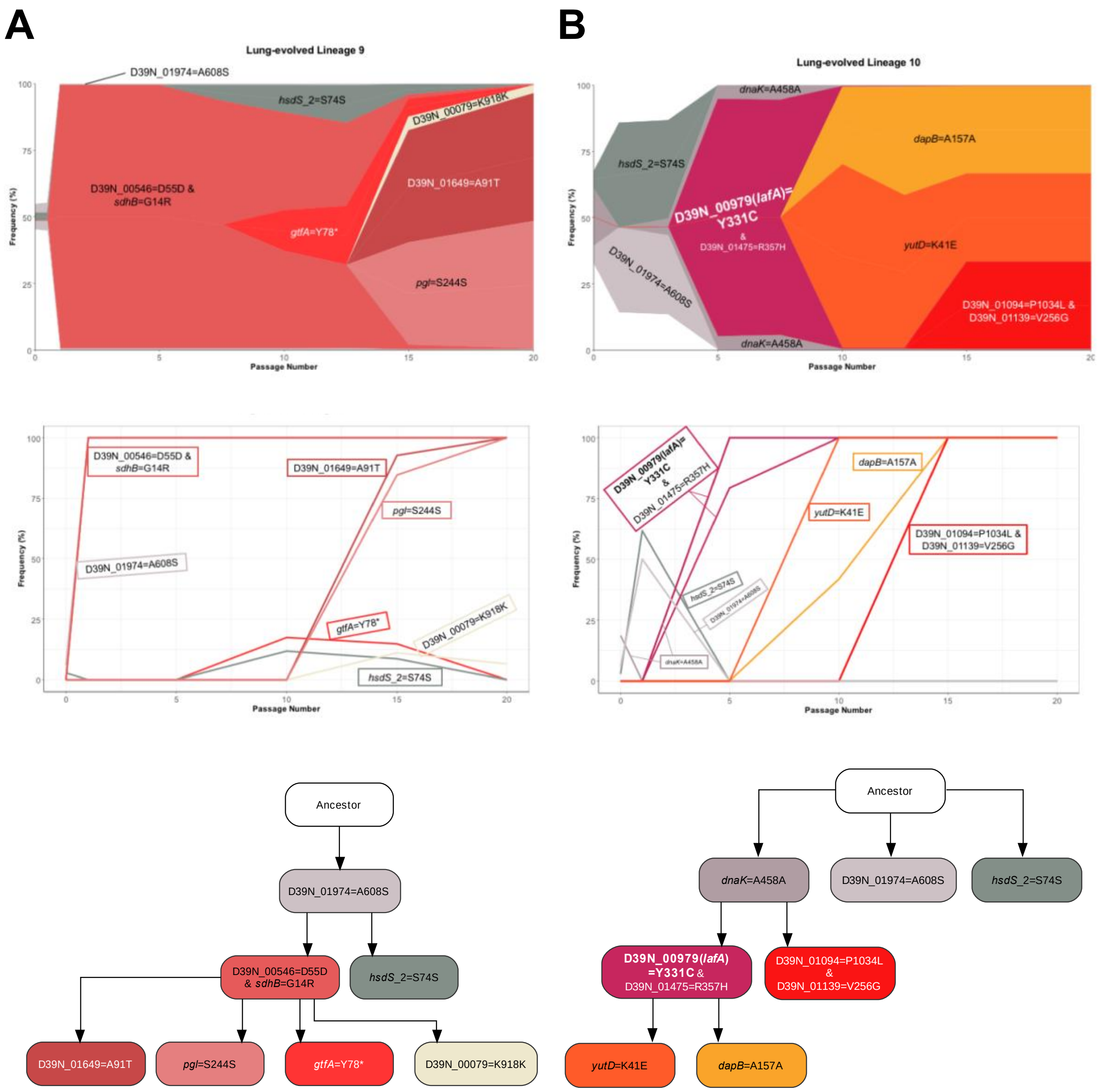

Supplementary Figure 11. Genotype emergence and dynamics in lung passaged pneumococcal populations. (A) Lung-passaged lineage 9. **(B)** Lung-passaged lineage 10. Panels, from top-to-bottom, are Muller plots, genotype frequency plots and genotype ancestry trees.

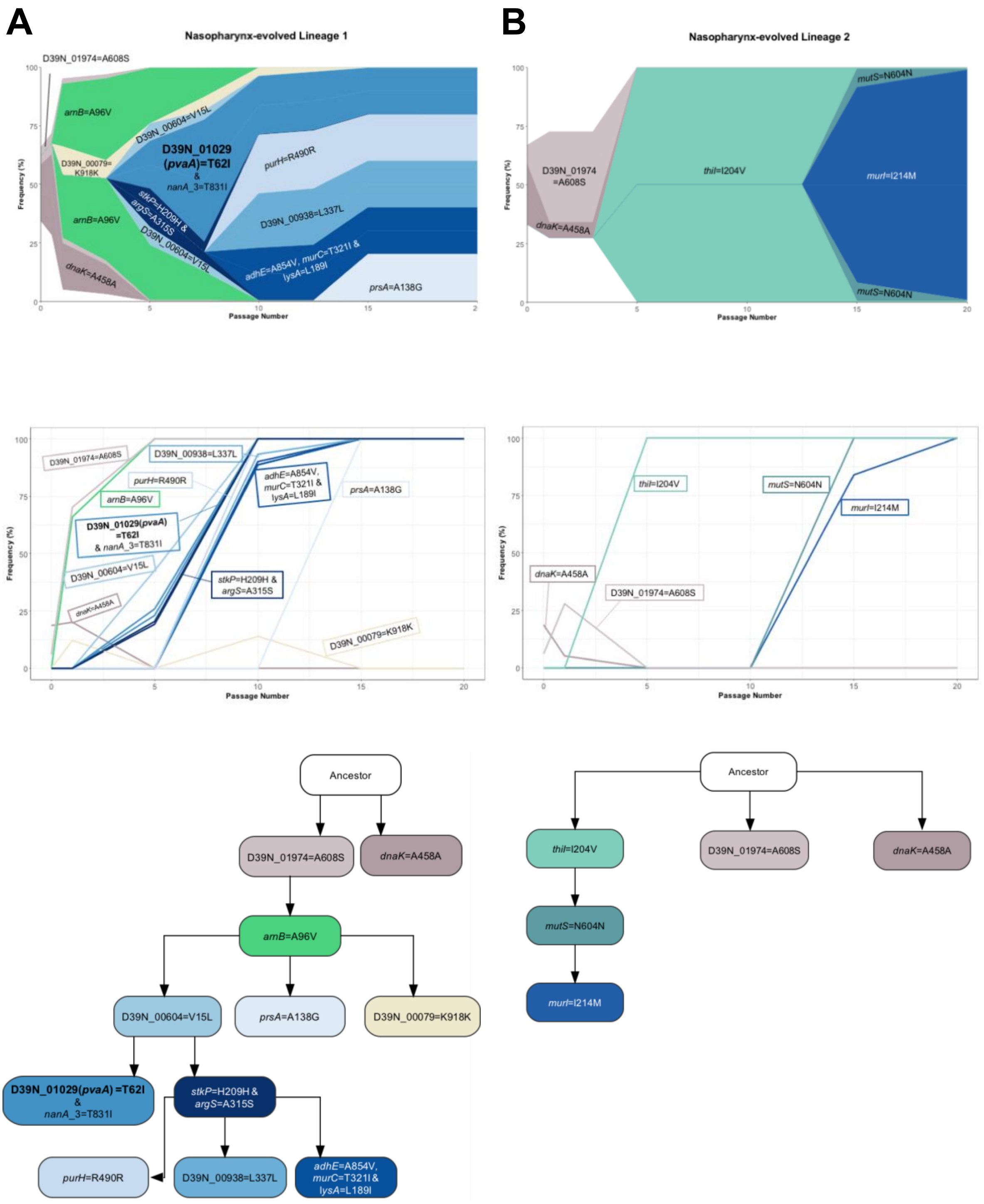

Supplementary Figure 12. Genotype emergence and dynamics in nasopharynx passaged pneumococcal populations. (A) Nasopharynx-passaged lineage 1. **(B)** Nasopharynx-passaged lineage 2. Panels, from top-to-bottom, are Muller plots, genotype frequency plots and genotype ancestry trees.

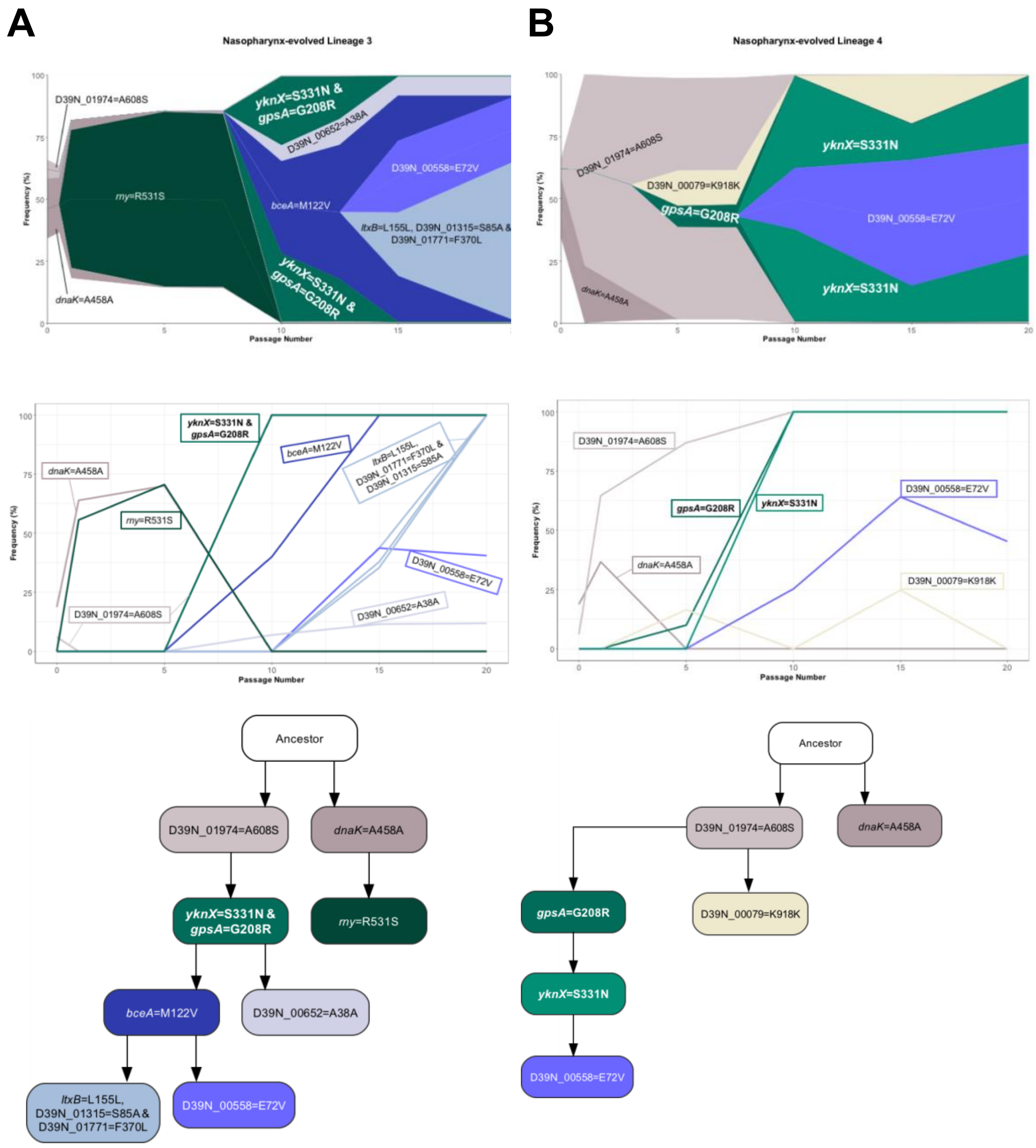

Supplementary Figure 13. Genotype emergence and dynamics in nasopharynx passaged pneumococcal populations. (A) Nasopharynx-passaged lineage 3. **(B)** Nasopharynx-passaged lineage 4. Panels, from top-to-bottom, are Muller plots, genotype frequency plots and genotype ancestry trees.

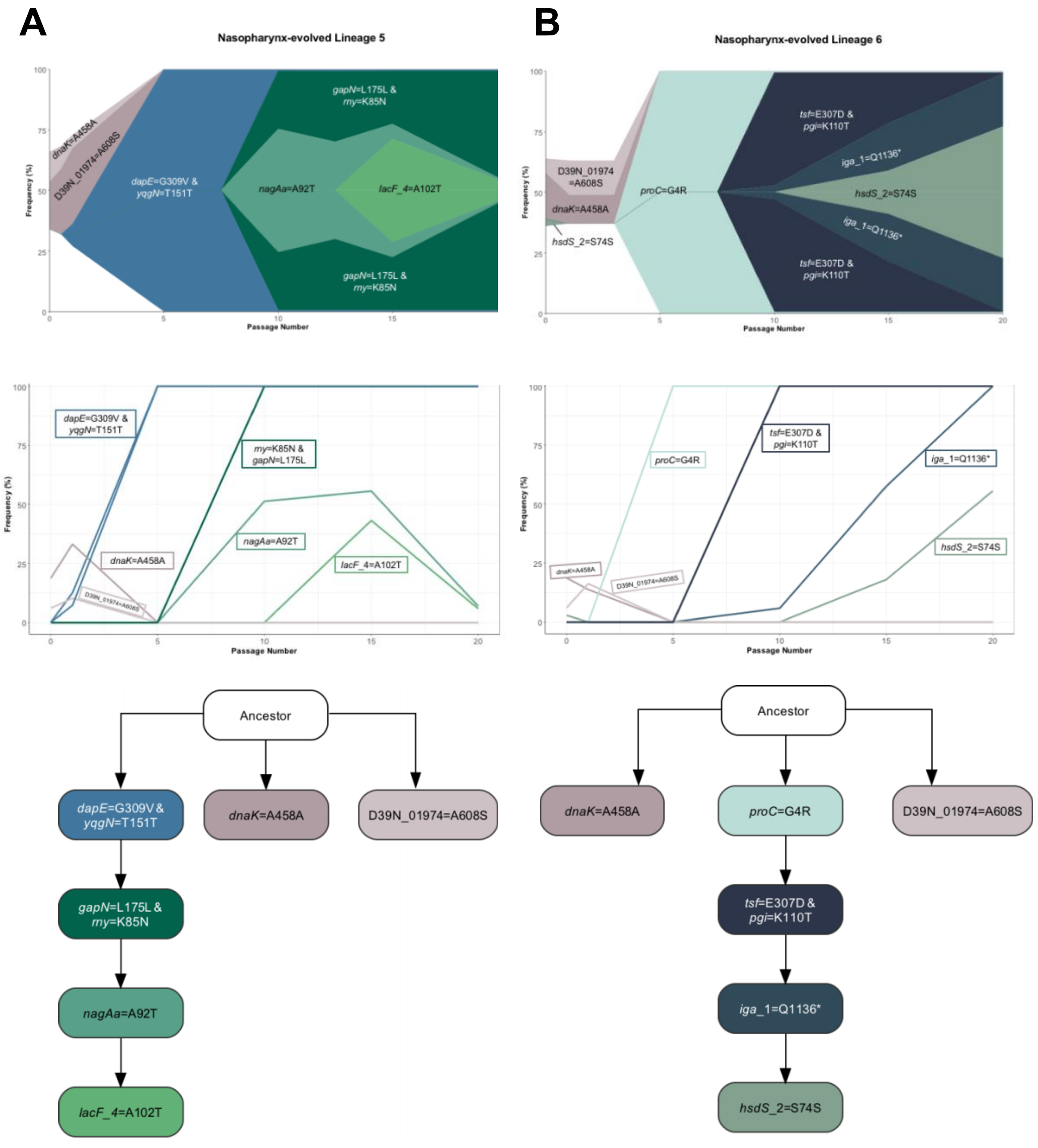

Supplementary Figure 14. Genotype emergence and dynamics in nasopharynx passaged pneumococcal populations. (A) Nasopharynx-passaged lineage 5. **(B)** Nasopharynx-passaged lineage 6. Panels, from top-to-bottom, are Muller plots, genotype frequency plots and genotype ancestry trees.

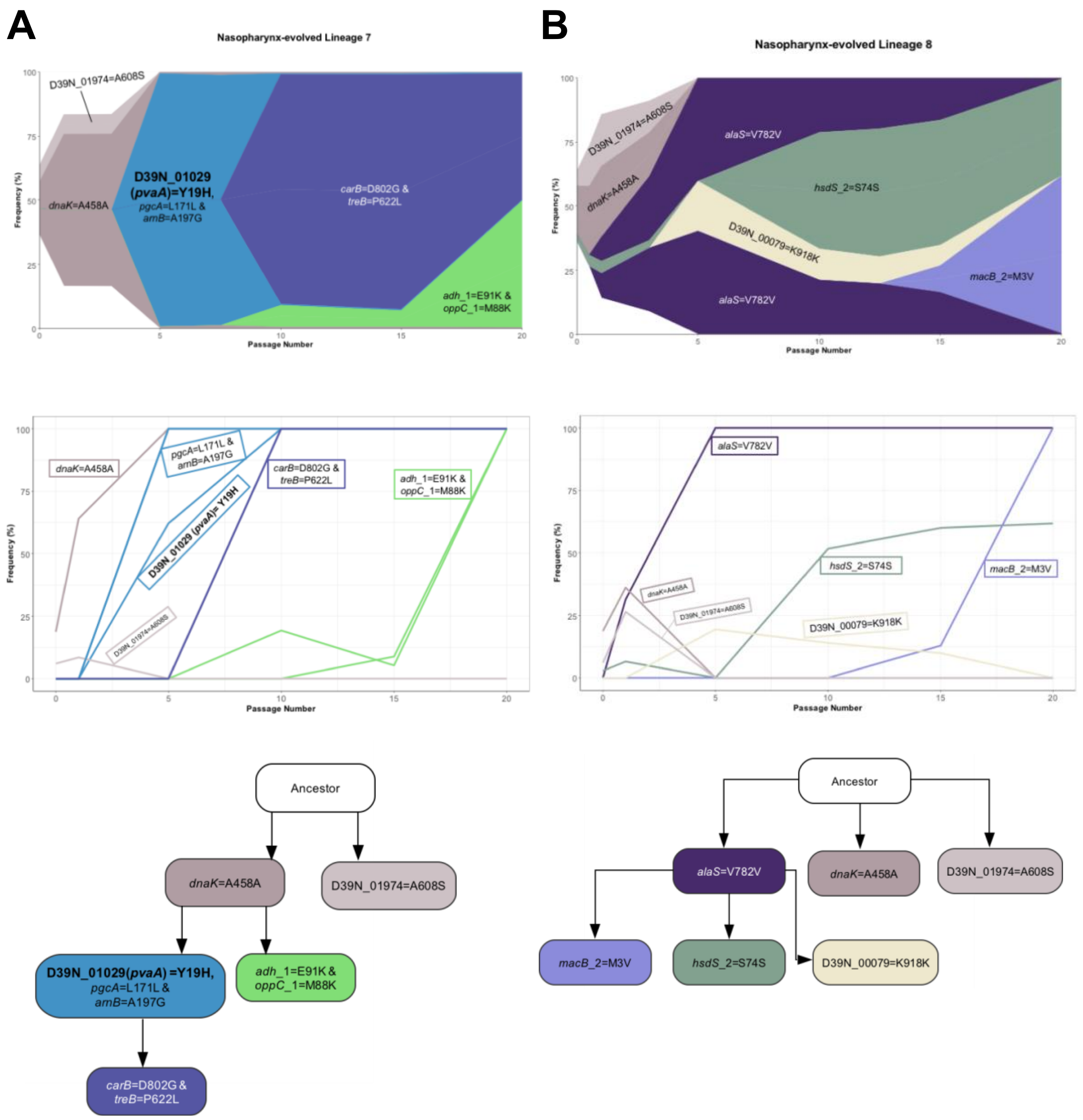

Supplementary Figure 15. Genotype emergence and dynamics in nasopharynx passaged pneumococcal populations. (A) Nasopharynx-passaged lineage 7. **(B)** Nasopharynx-passaged lineage 8. Panels, from top-to-bottom, are Muller plots, genotype frequency plots and genotype ancestry trees.

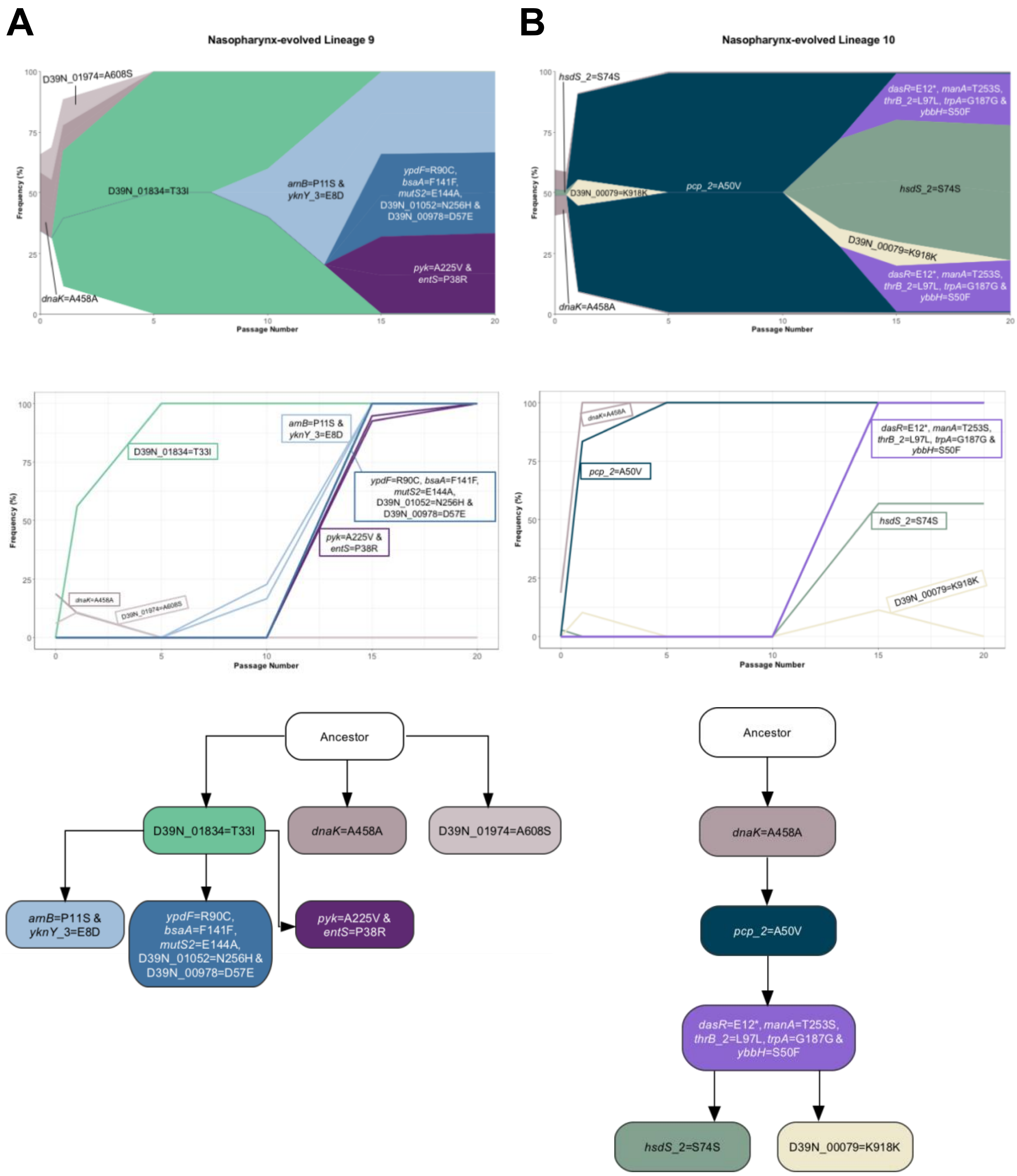

Supplementary Figure 16. Genotype emergence and dynamics in nasopharynx passaged pneumococcal populations. (A) Nasopharynx-passaged lineage 9. **(B)** Nasopharynx-passaged lineage 10. Panels, from top-to-bottom, are Muller plots, genotype frequency plots and genotype ancestry trees.

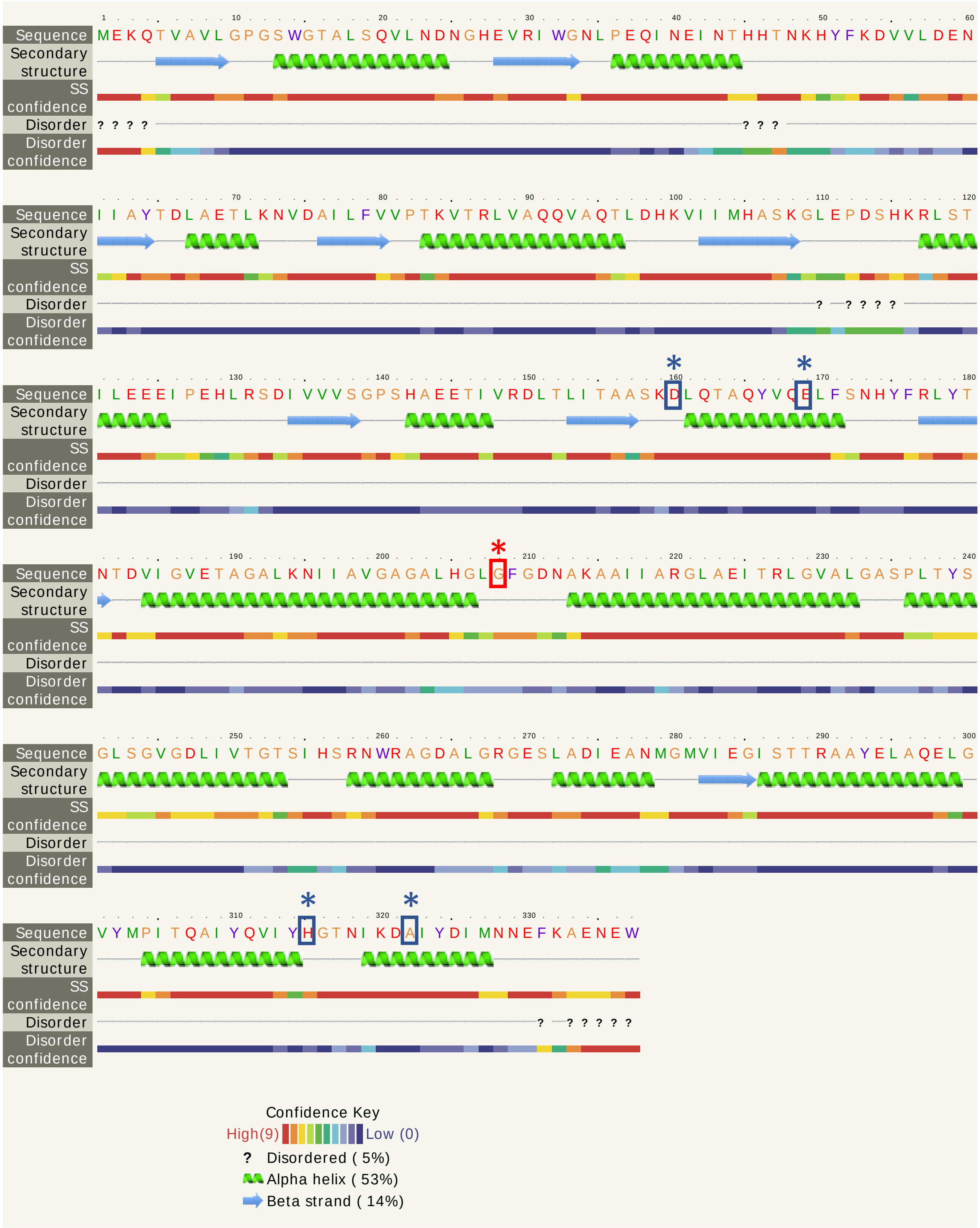

Supplementary Figure 17. Predicted secondary structure of pneumococcal glycerol-3-phosphate dehydrogenase. The D39 ancestor sequence is shown and the site of the G208R substitution identified in nasopharynx passaged lineages 3 and 4 is boxed in red. Identified substitutions in natural carriage of infants in the study of Chaguza *et al.* are boxed in blue. These were D160N, K169E (a glutamic acid is already present at this site in D39), H315Q and A322D. Image generated in Phyre2.

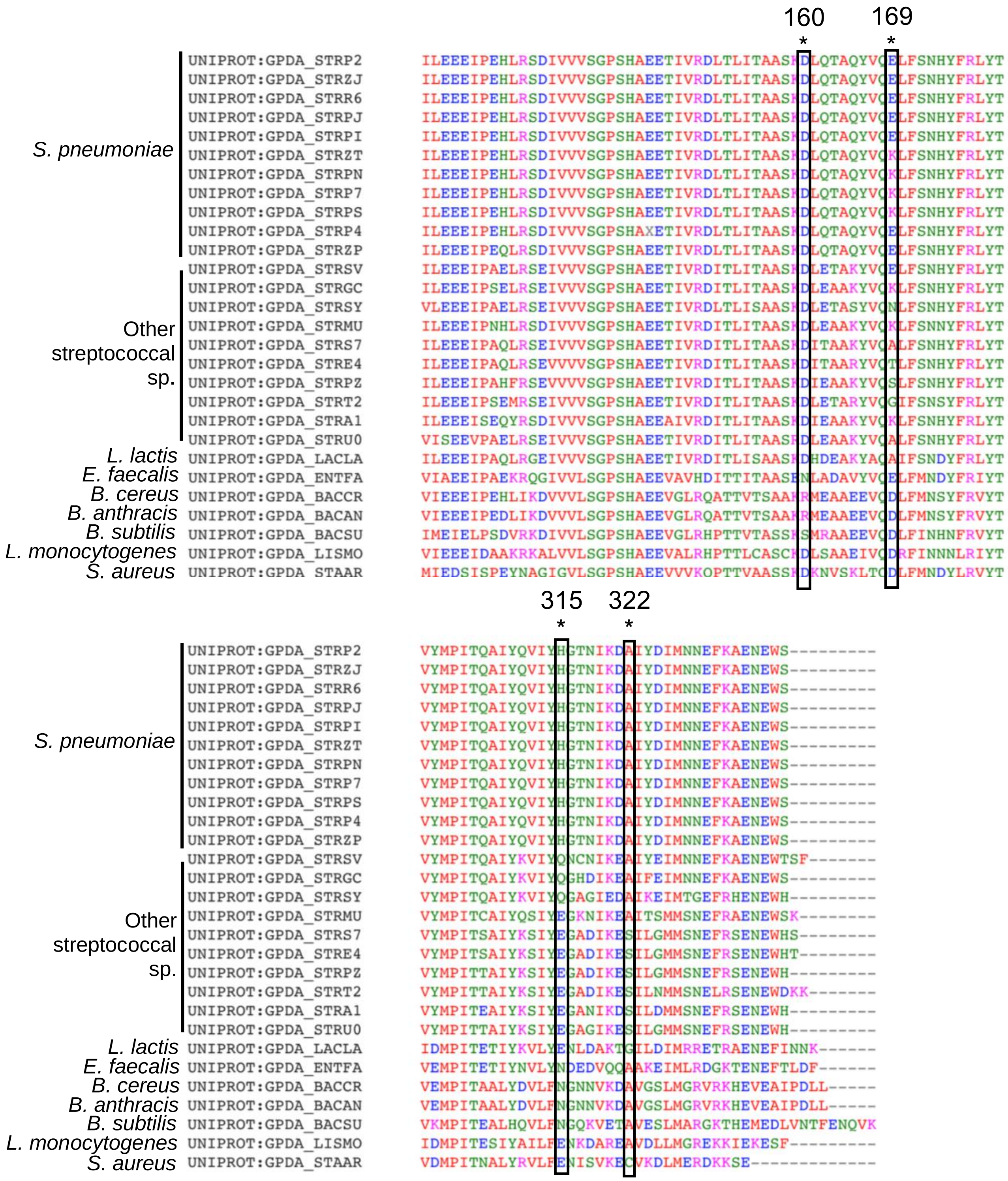

Supplementary Figure 18. Peptide sequence alignment of glycerol-3-phosphate dehydrogenase from streptococci and related species. The two regions of G3PDH in which amino acid substitutions were identified in natural colonisation of infants are shown, with the D39 ancestor at the top of the alignment. Positions of substitutions are boxed out in black. Streptococcal species included are (top to bottom) *S. pneumoniae, S. sanguinis, S. gordonii, S. suis, S. mutans, S. equi* subspecies zooepidemicus, *S. equi* subspecies equi, *S. pyogenes, S. thermophilus, S. agalactiae, S. uberis*. Amino acids are coloured by type: red = small, blue = acidic, magenta = basic, green = other. Image generated with Clustal Omega.

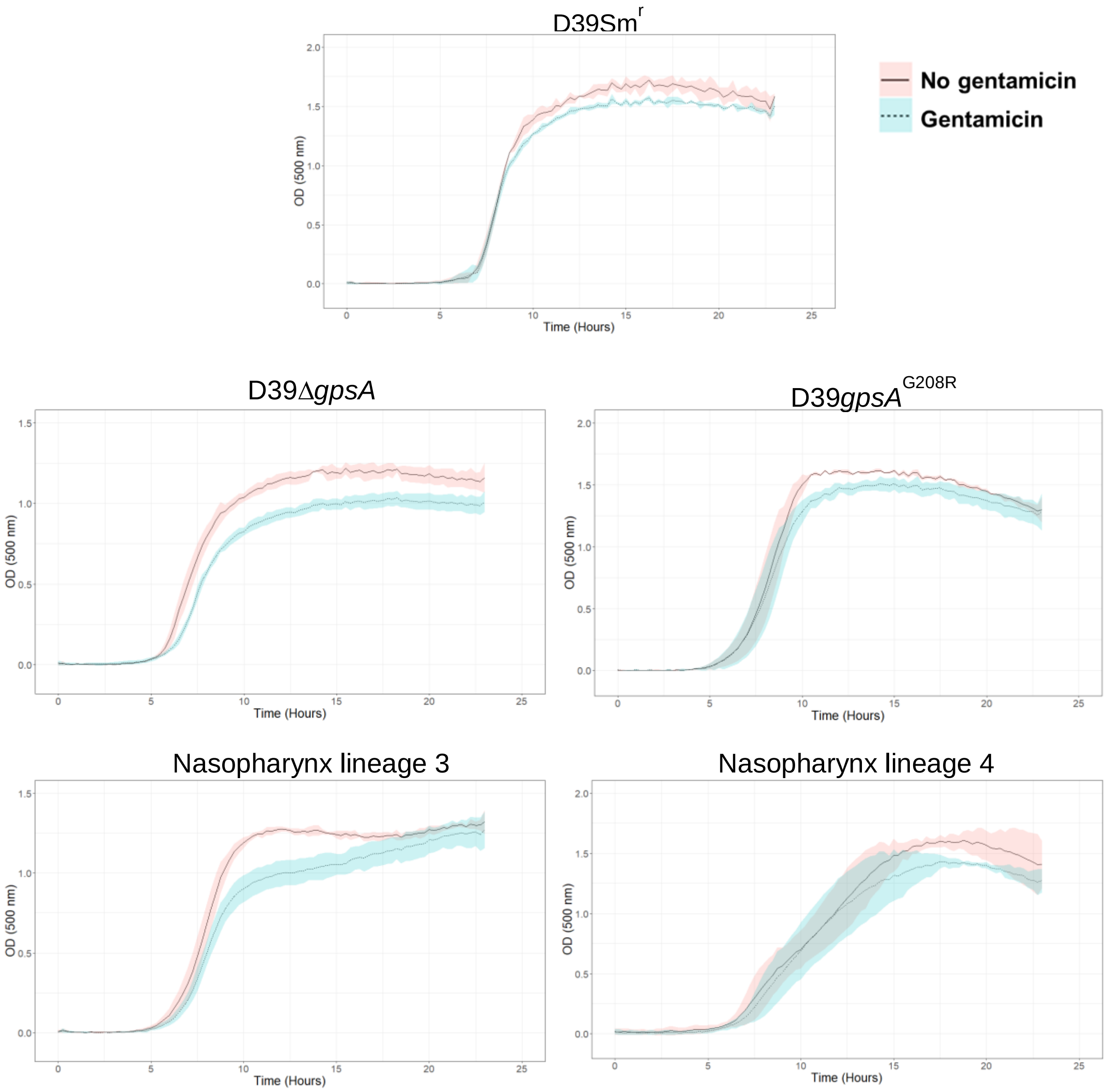

Supplementary Figure 19. Growth of *gpsA* mutants on the D39 background in broth. The streptomycin resistant D39 control (D39Sm^r), the *gpsA* deletion strain (D39Δ*gpsA*), the SNP mutant (D39*gpsA*^{G208R}) and the two nasopharynx passaged lineages carrying the G208R substitution in GpsA were grown in BHI broth in the presence or absence of 1 µg/ml gentamicin. Lines show the mean OD_{500}, determined at 15 minute intervals, of three biological replicates, with standard deviation depicted by shading.

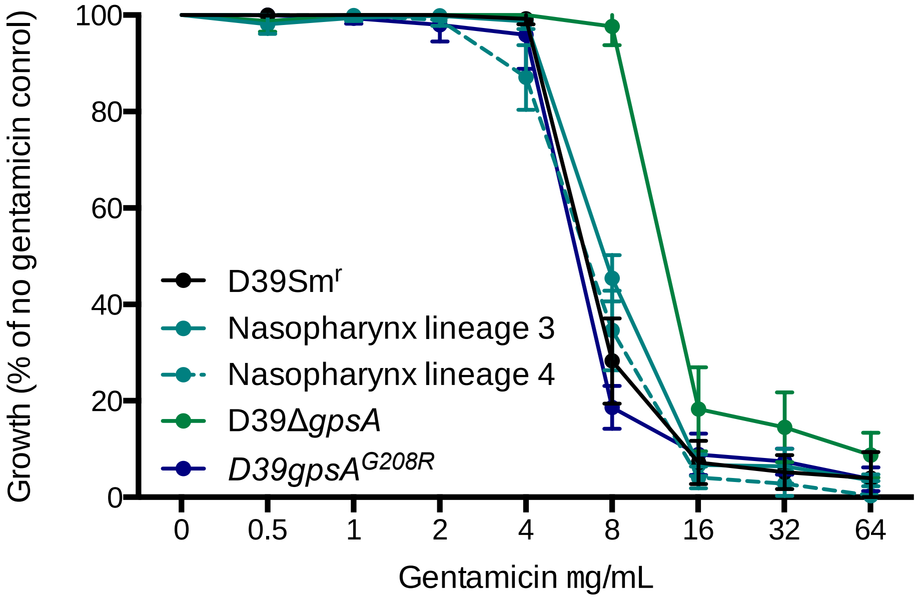

Supplementary Figure 20. Gentamicin resistance in D39 *gpsA* mutants. The streptomycin resistant D39 control (D39Sm^r), the *gpsA* deletion strain (D39Δ*gpsA*) and the SNP mutant (D39gpsA^{G208R}) were grown for 24 hours in BHI broth in the presence of two-fold increasing concentrations of gentamicin (range 0.5-64 μg/ml). Data are a composite of nine replicates from three independent experiments and show means with standard deviation. Growth is presented as a percentage of the growth achieved for each strain in the absence of gentamicin. MIC50 values were determined as 8 μg/ml (D39Sm^r, D39*gpsA*^{G208R}, nasopharynx lineages 3 and 4) and 16 μg/ml (D39Δ*gpsA*).

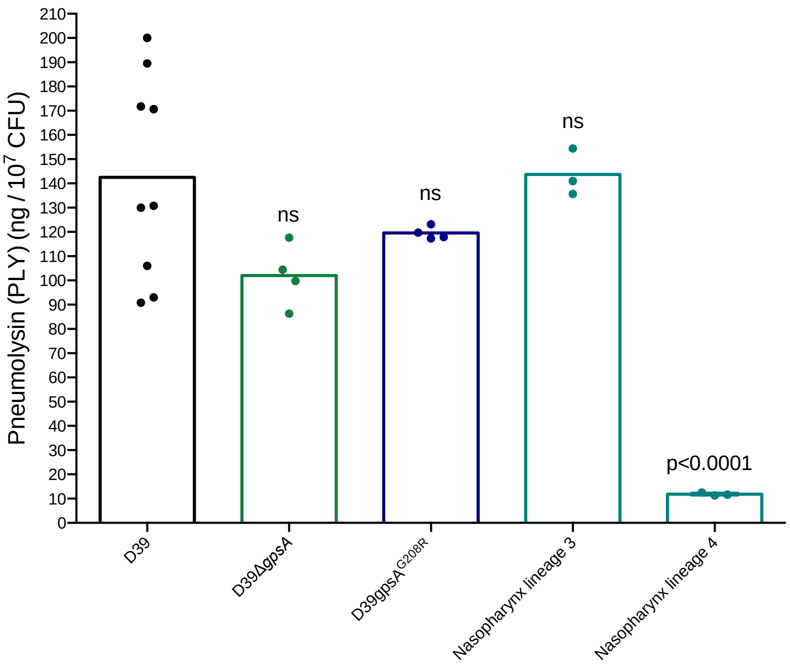

Supplementary Figure 21. Pneumolysin production is unaltered in *gpsA* mutants. ELISA-determined pneumolysin concentrations in lysates prepared from $1x10^7$ mid-log phase pneumococci. Concentrations were determined by comparison to a standard curve generated with purified pneumolysin. P-values are for comparisons with D39 from one-way ANOVA with Dunnett's multiple-comparisons test. Ns = not significant.

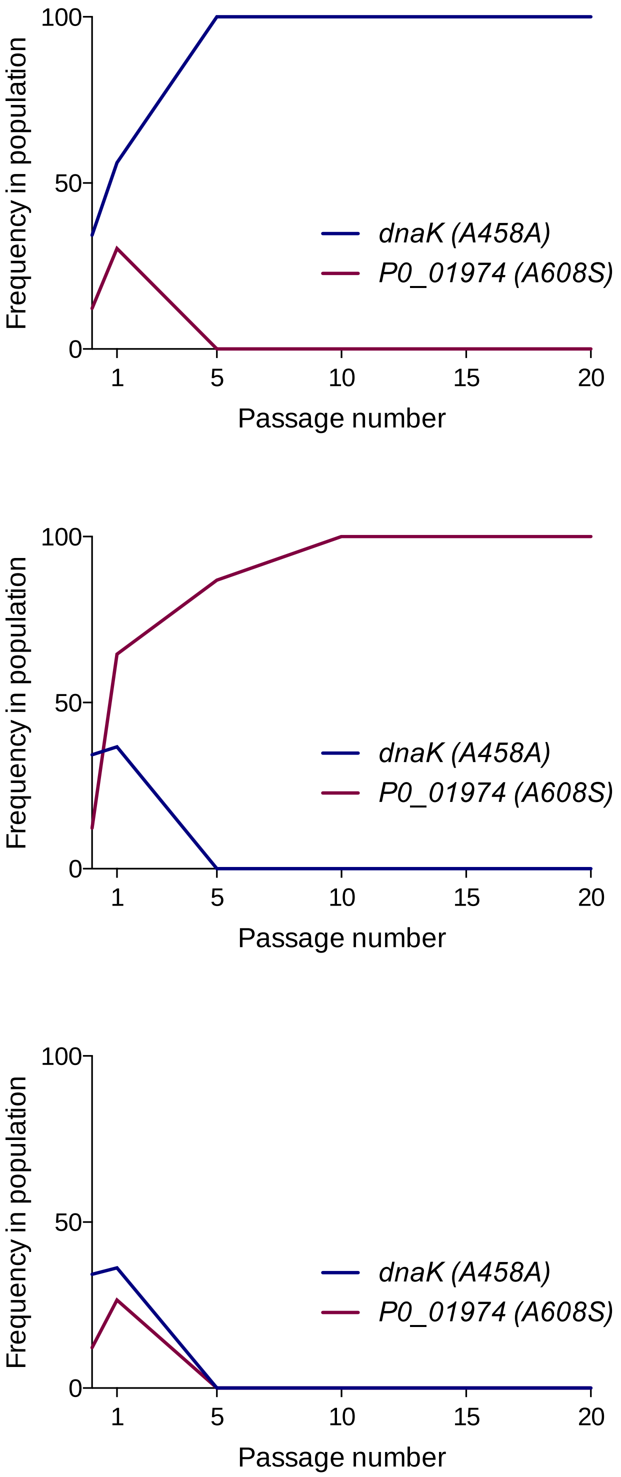

Supplementary Figure 22. Dynamics of mutations found in the ancestor population during experimental passage. Frequencies of synonymous *dnaK* mutation A458A and non-synonymous *P0_01974* mutation A608S over time during passage through nasopharynx and lungs. **(A)** Lung lineage 4, an example of a lineage where genotypes derived from the *dnaK* mutation-bearing portion of the ancestor drove the *P0_01974* mutation to extinction. **(B)** Nasopharynx lineage 4, an example of a lineage where *P0_01974* mutant-derived genotypes were successful at the expense of those derived from the *dnaK* mutant sub-population. **(C)** Nasopharynx lineage 8, an example of where both sub-populations were eradicated, suggesting another genotype within the ancestor population was successful.

Supplementary Table 1. Spontaneous mutation rates of pneumococcal lineages carrying mutations in DNA repair genes. Spontaneous rifampicin resistance mutants were quantified on antibiotic agar. Where no growth was observed on rifampicin plates (ancestor, lung lineage 10, nasopharynx lineage 8), the maximum possible mutation rate is indicated, based on the detection limit of the assay.

Pneumococcal population	DNA repair gene mutation	Spontaneous mutation rate
Ancestor	-	<8.57E-07
Lung lineage 3	*mutX1* (P29S)	5.02E-05
Lung lineage 6	*mutX1* (P29S)	4.52E-05
Lung lineage 10	-	<3.00E-07
Nasopharynx lineage 8	-	<7.32E-07
Nasopharynx lineage 9	*mutS2* (E144A)	2.12E-04

Supplementary Table 2. Gentamicin minimum inhibitory concentrations for D39 and experimentally evolved lineages. The minimum inhibitory concentration required to inhibit 50% of growth (MIC50) was determined in nutrient broth. The median MIC50 is shown, from 6 replicate cultures performed over two independent experiments. The range from individual replicates shown in brackets, in cases where variation was observed.

**Pneumococci**	**Gentamicin MIC50 (μg/ml)**
D39 Ancestor	8
Nasopharynx lineages	
1	8 (4-8)
2	8
3	8
4	8
5	8
6	8
7	8
8	4
9	4 (4-8)
10	8 (8-16)
Lung lineages	
1	8
2	8
3	8
4	8
5	8
6	8
7	8
8	8 (8-16)
9	8
10	8
Control lineages	
1	16 (4-16)
2	16
3	16 (8-16)
4	16
5	8
6	8
9	8 (8-16)
10	16

Supplementary Table 3. Pneumococci recovered from lungs during generation of nasopharynx-passaged lineages. Lung colony forming units of pneumococci recovered during experimental passage of pneumococci through nasopharynx, at day 7 post infection. Where there is no entry for a lineage/passage number, no pneumococci were recovered from lungs.

Lineage	Passage Number	Colony forming units/lung
1	6	3.30E+01
2	8	3.30E+01
2	17	1.67E+02
3	15	1.67E+02
4	5	3.33E+02
4	12	1.67E+04
5	4	1.67E+02
6	2	5.00E+02
6	5	3.30E+01
6	6	3.30E+01
7	10	1.67E+02
8	10	3.30E+01
8	12	3.33E+02
10	4	3.33E+02
10	13	3.33E+02
