# Supplementary material for: Pneumococcal Colonization and Virulence Factors Identified Via Experimental Evolution in Infection Models
Source: Mol Biol Evol. 2021 Jan 27;38(6):2209–26. doi: 10.1093/molbev/msab018 (PMC8136498; doi:10.1093/molbev/msab018)
Supplement: msab018_Supplementary_Data [file msab018_supplementary_data.zip › Supplementary Materials and Methods_Final.pdf]

Supplementary Materials and Methods

DNA extractions

Genomic DNA was extracted from fresh overnight cultures of pneumococcal isolates. A pre-lysis step was included where a pellet from 2 ml of overnight culture was incubated for 1 hour at 37°C with lysozyme (Sigma) and mutanolysin (Sigma) in TE buffer. Proteinase K (Qiagen) and Buffer AL (Qiagen) were then added and samples incubated at 56°C for 2 hours. The DNeasy Blood & Tissue Kit (Qiagen) was then used, following the manufacturer's instructions for purification of DNA from Gram-positive bacteria. Quality assessment of DNA was carried out by measuring the absorbance ratios 260/280 nm and 260/230 nm using a NanoDrop Spectrophotometer (Thermo Fisher Scientific); ratios of ≥1.8 were used as an indication of good quality DNA. A Qubit™ fluorometer system with the broad range DNA kit (Invitrogen) was used to quantify DNA, according to manufacturer's instructions. We required 10 µg of DNA at 200 ng/µl in a final volume of 50 µl for PacBio sequencing and for Illumina sequencing >1 µg of DNA between 20-150 ng/µl in a final volume of 25 µl was needed.

BLAST comparison of the D39 ancestor strain sequence annotation with D39V

In order to check for annotations of those genes labelled as hypothetical proteins in the Prokka (v1.13) annotated D39 ancestor genome, we compared all genes to those in the recently published assembly of the D39 strain(Slager, et al. 2018), using the command line standalone NCBI Nucleotide-Nucelotide BLAST programme (BLASTp, v2.9.0+)(Camacho, et al. 2009). Matches between genes in the two strains were determined by applying an E-value cut-off of 1E-40, and setting the best-hit filtering algorithm overhang parameter to 0.25. The results can be found in Supplementary Dataset 1.

Cluster of Orthologous Group (COG) functional annotation

The COG functional annotation(Tatusov, et al. 2000) of the D39 Prokka predicted CDS was carried out using the online eggNOG-mapper (v2) database using default settings(Huerta-Cepas, et al. 2019). The assigned COG categories of genes with all types of mutations found in the lung and nasopharynx passage 20 isolates were analysed to determine which COG functional groups were represented amongst the variant genes. The COG categories for those genes with mutations which appear in more than one lineage, are represented only once and gene variants also found in the control passage were removed from this analysis. The percentage of COG functional groups were determined and the analysis was represented as pie charts. The percentages are based on the total number of variant genes in the lung and nasopharynx passage 20 lineages.

Confirmation of SNPs in niche evolved lineages

DNA was extracted from the ancestor D39 strain and the lineages of interest as previously described. To confirm SNPs, the target gene was amplified via PCR, in a Veriti 96-well thermal cycler (Applied Biosystems), using the TaKaRa Ex Taq® DNA Polymerase kit (TakaraBio, Germany) with the PCR primers described in Supplementary Dataset 3 (primers were purchased from Eurofins Genomics). To confirm amplification of the relevant gene, PCR products were run using a 1% agarose gel in TAE buffer (Invitrogen). To extract and purify the PCR products from the gel, the MinElute Gel Extraction Kit (Qiagen) was used following manufacturer's instructions. The purified PCR products were quantified using the Nanodrop and sent for sequencing at Eurofins Genomics via the TubeSeq service. The resulting forward and reverse sequences were aligned using the MEGA alignment software (v10.1.5) against the relevant gene sequences to confirm the presence of the SNPs in the niche evolved lineages and the SNP absence in the D39 ancestor strain.

Construction of Muller plots, genotype frequency plots and genotype ancestry trees

The command line programme muller (v0.6.0 - https://pypi.org/project/muller/) with default parameters applied, was used to produce the genotypes and trajectories tables for each of the 20 nasopharynx and lung evolved lineages. These tables were then were used as inputs for ggplot2 (v3.3.2) and ggmuller (v0.5.4) in R-Studio (v4.0.2), to produce Muller plots, genotype frequency plots and genotype ancestry trees for each lineage. Genes which had variants in two or more lineages were manually coloured the same across the different plots. The genotypes found in the ancestral strain were coloured as white in each lineage Muller plot, except where Breseq assigned the *dnaK* mutation or *P0_01974* ancestral SNPs into other genotypes.

Visualising the non-synonymous mutations on the D39 ancestor genome

The Circa software (OMGenomics, http://omgenomics.com/circa/) was used to construct Circos plots for visualisation of lineage SNPs on the D39 ancestor genome. The Prokka generated GFF file was converted to a tab-separated format that indicated the start and end positions and names of the 2069 D39 ancestor genes, these are displayed as grey lines on the 1st track of the genome maps. The positions of the genes containing non-synonymous SNPs, identified in twenty-times passaged pneumococci from the control passage or from lung and nasopharynx, were drawn on the maps with circles.

Analysing growth dynamics of pneumococcal isolates

Pneumococcal isolates were streaked onto gentamycin BAB plates and incubated overnight at 37°C in 5% CO_2. A loopful of colonies were resuspended into BHI and 200 µl of culture

with a starting OD of approximately 0.002 at 500 nm, was added to the relevant wells of a 96 well plate (Corning Laboratories, Corning, NY). BHI broth was used as a negative control to make sure there was no contamination. The plate was incubated in a Varioskan multimode microplate reader (TheremoFisher Scientific) at 37 ˚C with 5% CO_2 for 24 hours, taking reads at 500 nm every 15 mins with double orbital shaking for 10 seconds before the reading. The mean OD readings from the negative control wells were subtracted from the test wells and the resulting OD readings were analysed using the R package GrowthCurver (v 0.3.1) and isolate growth dynamics were determined and represented as the area under the logistic curve (AUC). This protocol was repeated with freshly streaked plates to obtain biological replicates. Growth curves were plotted using the R programme ggplot2 (v3.3.2) and a shaded area was included to represent the standard deviation of the biological replicates at every OD reading. The effect of gentamicin on growth was assessed by addition of 1 µg/ml gentamicin to cultures, with bacteria-only controls included in all assays. The concentration of 1 µg/ml was chosen to match the concentration in agar, to which pneumococci were exposed during experimental passage.

Mutant construction

The D39 ancestor strain was first made resistant to streptomycin by introducing a point mutation in the *rpsL* gene to produce a K56T variant. A SOEing PCR using the TaKaRa Ex Taq® DNA Polymerase kit (TakaraBio, Germany) was performed with the streptomycin resistant up_fragment and down_fragment primers (forward and reverse) (all primer sequences can be found in Supplementary Dataset 3). The D39 ancestor strain was then transformed using the resulting SOEing PCR product by growing the strain at 37°C with 5% CO_2 in C+Y media. Once an OD of 0.07-0.1 (500 nm) was reached, 1 ml was transferred to a 1.5 ml tube and 3 µl competence stimulating peptide (CSP)1 was added along with the PCR product. This was incubated for 3 hours at 37°C with 5% CO_2. Mutants were selected on TSA + 3% blood + 20 ug/mL neomycin + 800 ug/mL streptomycin. The resulting streptomycin resistant strain was then used for subsequent mutant construction. Targeted deletion of the *gpsA* (D39Δ*gpsA*) was carried out using the sweet Janus system. The sweet Janus cassette was amplified using the SweetJanus forward and reverse primers. The flanking regions upstream and downstream of the *gpsA* locus (~1 kb) were amplified to produce the PCR products GpsA_Up_SJ and GpsA_Down_SJ. A SOEing PCR product was produced by combining both the flanking region and the Sweet Janus PCR products. To delete *gpsA*, the resulting SOEing PCR product was used to transform the D39 streptomycin resistant ancestor strain described above. The mutants were selected by plating 200 µl of transformation product on TSA + 3% blood + 20 µg/mL neomycin + 400 µg/mL kanamycin. Selected mutants were also plated onto TSA + 3% blood + 20 µg/mL neomycin + 800 µg/mL

streptomycin + 10% sucrose to confirm loss of streptomycin resistance. To create the G208R point mutation in the *gpsA* gene, the desired gene region was amplified to swap into the locus site previously deleted in D39Δ*gpsA*. The PCR parts GpsA_G208R_Up and GpsA_G208R_Down were produced using combinations of the R primer (for upstream) and F primer (for downstream) with overlapping sequences of the *gpsA* point mutation (Supplementary Dataset 3). A SOEing PCR product was produced by combining these PCR parts and the resulting product was used to transform D39Δ*gpsA* as previously described. The *gpsA* point mutation mutants were selected on TSA + 3% blood + 20 µg/mL neomycin + 800 µg/mL streptomycin + 10% sucrose, no growth on kanamycin was also verified. To confirm successful transformation of the knockout and the point mutation mutants, primers outside of the transformed regions were used for PCR and the region was subsequently sequenced.

Cell lines

Human lung adenocarcinoma epithelial cell line A549 (ATCC CCL-185) was used to assess adhesion and invasion potential of pneumococci. All cells were tested for mycoplasma contamination prior to starting experiments and confirmed as negative. A549 cells were maintained in Dulbecco's Modified Eagle Medium (DMEM) supplemented with 10% Foetal Bovine Serum (FBS).

Adhesion/Invasion assays

Overnight cultures of pneumococci were diluted to an OD of 0.4 at 500 nm and centrifuged at 4000 rpm for 10 min. The pellet was resuspended in DPBS (pH 7.4) and diluted in DMEM supplemented with 5% FBS, for infection of A549 airway epithelial cell monolayers at a multiplicity of infection (MOI) of 10. The plates were centrifuged at 800 rpm for 5 min to synchronise infection and incubated at 37ºC for 1 hour. Following the 1 hour infection, the media was removed from the wells which were then washed 4X with DPBS. After washing, in the wells of the plate designated for assessment of adhesion, 100 µl/well of Trypsin + 0.025% EDTA was added and incubated for 5-10 minutes at 37°C to detach the cells. The cells were then lysed by adding 1 ml/well of 0.025% Triton X-100 diluted in DMEM+FCS and incubated for 10 minutes at 37°C. To determine the cfu/ml of bacteria which had adhered to the A549 cells, samples were serially diluted 1:10 in PBS and 60 µL was plated on gentamycin BAB plates and incubated 37°C in 5% CO_2. For the plates designated invasion, after washing, the monolayers were incubated with DMEM+FBS containing penicillin (10 µg/ml) and gentamicin (400 µg/ml) for 2 h to kill extracellular pneumococci. Following this incubation, cells were lysed and bacteria enumerated as described above. The percentage

of pneumococci that adhered/invaded was calculated as (CFU in the lysate / CFU used for infection)×100.

Pneumolysin Detection ELISA

Ninety-six-well ELISA microplates (Corning Laboratories, Corning, NY) were coated overnight at 4°C with 1 µg in 100 µl per well mouse anti-Ply (PLY-4) antibody (Abcam, ab71810). After washing 5 times with 300 µl/well of wash buffer (PBS + 0.05% tween), plates were blocked for 3 hours with 200 µl/well PBS + 20% FBS. Pneumococci were prepared from overnight cultures that were centrifuged for 15 mins at 3000 rpm and the pellet resuspended in 10 ml PBS and lysed with the addition of 100 µl of sodium deoxycholate at 10%. This was incubated at room temperature for 10 minutes, centrifuged for 15 minutes at 3000 rpm and the supernatant used for the assay. For the standards, a ten 2-fold pneumolysin toxin dilution series was prepared. After washing the plate, the bacterial lysates and standards at 100 µl/well were added and incubated at room temperature for 2 hours. After another wash, 1 µg/well rabbit anti-Ply antibody (Abcam, ab71811) in 100 µl of diluent was added and incubated for 2 hours. Plates were washed, and goat anti-rabbit–alkaline phosphatase antibody (Abcam, ab97048) was added for 30 minutes at room temperature. After washing, 100 µl/well pNPP colour reagent (Sigma) was added for 30 minutes and incubated in the dark before the reaction was stopped with 100 µl of 1m NaOH. The absorbance was measured at 405 nm in a Varioskan multimode microplate reader (TheremoFisher Scientific).

Protein Sequence Comparisons

Phyre^2 (Kelley, et al. 2015) was used to generate protein secondary structure predictions for the ancestor D39 glycerol-3-phosphate dehydrogenase. For alignment comparison, Clustal Omega at EMBL-EBI (Goujon, et al. 2010; Sievers, et al. 2011) was used, with D39 ancestor G3PDH protein sequence used as the input. From the resulting BLAST alignment, all available pneumococcal sequences were selected, along with one entry for each of the other available streptococcal species and single entries of other bacterial species with >50% protein identity.

Gentamicin Minimum Inhibitory Concentration Assays

Two-fold serial dilution of gentamicin were prepared in BHI in 96-well plates, covering a range of 64 to 0.5 µg/ml. Pneumococci from frozen stocks were standardised to OD_{500} 0.002 in BHI and were plated in triplicate and incubated for 20 hours at 37°C. The minimal inhibitory concentration was determined by measuring absorbance with an Fluostar Omega

Microplate Reader (BMG Labtech) and calculating percentage reduction in comparison to the untreated growth control for each isolate.

Spontaneous Mutation Frequency Assessment

Pneumococcal isolates were grown to an OD_{600} of 0.4 in BHI serum broth and 100 µl was plated onto BAB supplemented with 5% defibrinated horse blood and 0.125 µg/ml rifampicin (Sigma). Spontaneously resistant colonies were enumerated after incubation for 24 hours at 37°C in 5% CO_2. In parallel, a serial dilution of the OD_{600} 0.4 cultures was carried out and plated onto BAB plates containing no antibiotics, to determine the bacterial numbers per 100 µl. The spontaneous mutational frequency was calculated by dividing the CFU per 100 µl in rifampicin plates by the CFU per 100 µl on antibiotic-free plates.

References

Camacho C, Coulouris G, Avagyan V, Ma N, Papadopoulos J, Bealer K, Madden TL. 2009. BLAST+: architecture and applications. BMC Bioinformatics 10:421.

Goujon M, McWilliam H, Li W, Valentin F, Squizzato S, Paern J, Lopez R. 2010. A new bioinformatics analysis tools framework at EMBL-EBI. Nucleic Acids Res 38:W695-699.

Huerta-Cepas J, Szklarczyk D, Heller D, Hernandez-Plaza A, Forslund SK, Cook H, Mende DR, Letunic I, Rattei T, Jensen LJ, et al. 2019. eggNOG 5.0: a hierarchical, functionally and phylogenetically annotated orthology resource based on 5090 organisms and 2502 viruses. Nucleic Acids Res 47:D309-D314.

Kelley LA, Mezulis S, Yates CM, Wass MN, Sternberg MJ. 2015. The Phyre2 web portal for protein modeling, prediction and analysis. Nat Protoc 10:845-858.

Sievers F, Wilm A, Dineen D, Gibson TJ, Karplus K, Li W, Lopez R, McWilliam H, Remmert M, Soding J, et al. 2011. Fast, scalable generation of high-quality protein multiple sequence alignments using Clustal Omega. Mol Syst Biol 7:539.

Slager J, Aprianto R, Veening JW. 2018. Deep genome annotation of the opportunistic human pathogen Streptococcus pneumoniae D39. Nucleic Acids Res 46:9971-9989.

Tatusov RL, Galperin MY, Natale DA, Koonin EV. 2000. The COG database: a tool for genome-scale analysis of protein functions and evolution. Nucleic Acids Res 28:33-36.
